# Competition between resident rhizosphere bacteria enhances phytopathogen suppression via emergent antagonism

**DOI:** 10.1093/ismejo/wrag229

**Published:** 2026-09-04

**Authors:** Yue Yin, Thomas Pommier, Xiaoni Zhang, Fang Chi, Ziru Zhang, Connor G Hendrich, Emma K Sheriff, Chujin Ruan, Yangchun Xu, Ville-Petri Friman, Zhong Wei

**Affiliations:** Jiangsu Provincial Key Lab for Organic-based Fertilizer Creation and Soil Health Manipulation, Key Lab of Organic-based Fertilizers of China, Jiangsu Collaborative Innovation Center for Solid Organic Wastes, Educational Ministry Engineering Center of Resource-saving Fertilizers, College of Resources and Environmental Sciences, Nanjing Agricultural University, Nanjing 210095, China; Department of Microbiology, University of Helsinki, Viikinkaari 9, Helsinki 00014, Finland; Jiangsu Provincial Key Lab for Organic-based Fertilizer Creation and Soil Health Manipulation, Key Lab of Organic-based Fertilizers of China, Jiangsu Collaborative Innovation Center for Solid Organic Wastes, Educational Ministry Engineering Center of Resource-saving Fertilizers, College of Resources and Environmental Sciences, Nanjing Agricultural University, Nanjing 210095, China; Department of Research, Development and Innovation, Setec Energie Environnement, 97-101 bvd Marius Vivier-Merle, Lyon 69003, France; School of Environmental Science and Engineering, Sun Yat-sen University, Guangzhou 510275, China; Department of Microbiology, University of Helsinki, Viikinkaari 9, Helsinki 00014, Finland; Jiangsu Provincial Key Lab for Organic-based Fertilizer Creation and Soil Health Manipulation, Key Lab of Organic-based Fertilizers of China, Jiangsu Collaborative Innovation Center for Solid Organic Wastes, Educational Ministry Engineering Center of Resource-saving Fertilizers, College of Resources and Environmental Sciences, Nanjing Agricultural University, Nanjing 210095, China; Department of Microbiology, University of Helsinki, Viikinkaari 9, Helsinki 00014, Finland; Department of Microbiology, University of Helsinki, Viikinkaari 9, Helsinki 00014, Finland; College of Ocean and Earth Sciences, Xiamen University, Xiamen 361102, China; Jiangsu Provincial Key Lab for Organic-based Fertilizer Creation and Soil Health Manipulation, Key Lab of Organic-based Fertilizers of China, Jiangsu Collaborative Innovation Center for Solid Organic Wastes, Educational Ministry Engineering Center of Resource-saving Fertilizers, College of Resources and Environmental Sciences, Nanjing Agricultural University, Nanjing 210095, China; Jiangsu Provincial Key Lab for Organic-based Fertilizer Creation and Soil Health Manipulation, Key Lab of Organic-based Fertilizers of China, Jiangsu Collaborative Innovation Center for Solid Organic Wastes, Educational Ministry Engineering Center of Resource-saving Fertilizers, College of Resources and Environmental Sciences, Nanjing Agricultural University, Nanjing 210095, China; Department of Microbiology, University of Helsinki, Viikinkaari 9, Helsinki 00014, Finland; Jiangsu Provincial Key Lab for Organic-based Fertilizer Creation and Soil Health Manipulation, Key Lab of Organic-based Fertilizers of China, Jiangsu Collaborative Innovation Center for Solid Organic Wastes, Educational Ministry Engineering Center of Resource-saving Fertilizers, College of Resources and Environmental Sciences, Nanjing Agricultural University, Nanjing 210095, China

**Keywords:** bacterial pairwise interactions, asymmetric competition, emergent antagonism, biocontrol, rhizosphere microbiome

## Abstract

Resident bacterial interactions can shape the invasion resistance of rhizosphere microbiomes, but whether interactions between weakly antagonistic resident bacteria can generate emergent antagonism against invading pathogens remains poorly understood. Here, we used a systematic pairwise interaction screening to identify *Ralstonia pickettii* RAL5 and *Acinetobacter oleivorans* ACI4 bacterial pair, that together provided a strong suppression of the phytopathogenic *Ralstonia solanacearum* Rs1115 strain. Although RAL5 and ACI4 monocultures only weakly inhibited pathogen growth, the RAL5–ACI4 co-cultures strongly suppressed the Rs1115, which was associated with asymmetric competition where the RAL5 dominated the ACI4 species. In line with this competitive asymmetry, broad transcriptional reprogramming was detected in RAL5 and only limited stress- and catabolism-associated responses in ACI4. The increased suppressiveness of co-cultures was associated with clear shifts in the extracellular metabolite profile, including the accumulation of candidate antimicrobial metabolites (e.g. a novobiocin-like feature and 4-aminophenol), and with the release of intracellular contents from ACI4 following RAL5-mediated lysis. The observed emergent antagonism also held in greenhouse experiments with tomato, where the RAL5–ACI4 consortium reduced *R. solanacearum* abundance and bacterial wilt severity relatively much more compared to when either strain was applied alone. Together, these results suggest that competitive interactions between resident bacteria can activate latent biocontrol potential in rhizosphere microbiomes, providing a new approach to harness resident bacterial interactions for enhanced pathogen suppression and biocontrol.

## Introduction

Soil-borne pathogens such as *Ralstonia solanacearum* threaten sustainable agriculture and cause substantial economic losses worldwide [1]. The rhizosphere microbiome serves as a direct barrier to pathogen invasion and forms the first line of defense against soil-borne pathogens [2, 3]. Deciphering the mechanisms by which bacterial communities resist pathogen invasion is hence fundamental to managing plant diseases and improving agricultural productivity. At the level of individual taxa, resident bacteria can restrict pathogen proliferation and invasion through multiple mechanisms, including competition for nutrients and iron, secretion of antimicrobial compounds, and induction of plant host systemic resistance [4–6]. Beyond species-level traits, also community-level properties such as community diversity, functional complementarity, and trophic network structure can shape the invasion resistance of plant-associated microbiomes against pathogens [7–9]. Such community-level effects arise not only as the sum of the individual activities, but also from cooperative (facilitative) and competitive (antagonistic) interactions among resident rhizosphere community members, which can either strengthen or weaken the community-level invasion resistance [10–12].

Resident community member interactions are predicted to influence invasion resistance through distinct ecological processes. Cooperative interactions can contribute to invasion resistance by promoting resource partitioning and metabolic complementarity among resident species [13, 14]. These interactions may also support resident niche occupation through biofilm formation and metabolite exchange, thereby restricting the space and resources available for pathogen establishment [14–16]. Cooperative communities, however, may become less stable when species rely on their partners for particular functions or when selection favors social cheats that benefit disproportionately from cooperative traits, thereby eroding cooperation over time [17, 18]. Theoretical work also predicts greater stability in competitive than cooperative communities under some conditions [19]. Experimental studies further show that antagonistic interactions among resident bacteria can restrict pathogen invasion both in vitro and in the tomato rhizosphere [20]. Such invasion resistance can arise through direct pathogen inhibition, consistent with the higher prevalence of antimicrobial biosynthetic genes detected in competitive than in cooperative microbial consortia [13]. Competitor identity has been shown to determine whether antibiotic production is induced or reduced during competition [21, 22]. In several well-characterized model taxa (*Bacillus, Streptomyces*, and *Pseudomonas*), specific competitive interactions activate otherwise silent biosynthetic gene clusters (BGCs) and increase the production of siderophores, antibiotics, and other secondary metabolites, thereby indirectly reinforcing pathogen suppression [23–26]. When such interactions generate stronger inhibition of a target organism than either strain produces alone, the resulting phenotype represents emergent antagonism. Current understanding of emergent antagonism remains largely gene-centered and has been restricted to a limited set of model taxa with known biosynthetic capacity. Prioritizing strains with recognizable antimicrobial genes or BGCs may bias discovery toward established antimicrobial producers, whereas monoculture screening may overlook suppressive functions that become evident only in the presence of other microbial species. Interaction-based screening can therefore broaden candidate selection beyond isolates with strong constitutive inhibition or recognizable antimicrobial biosynthetic potential by identifying suppressive activity that emerges only in particular strain pairings. However, it remains unclear how frequently such emergent antagonism occurs among rhizosphere bacterial taxa outside classical antibiotic producers. Also, more research is needed to understand the underlying molecular mechanisms of interaction-dependent emergent antagonism and if it is relevant in natural plant rhizosphere microbiomes, potentially affecting plant protection and pathogen biocontrol.

To address these questions, we used a defined collection of tomato rhizosphere isolates to test if pairwise resident–resident community member interactions can generate emergent antagonism against the phytopathogenic *R. solanacearum* bacterium, and whether such effects translate into disease suppression under greenhouse conditions (Fig. 1). The use of non-pathogenic congeners or avirulent strains of phytopathogens is an established biocontrol strategy because shared ecological requirements may enable them to occupy overlapping niches and compete for similar resources [27–29]. Here, we selected the tomato rhizosphere isolate *Ralstonia pickettii* RAL5, a non-pathogenic congener of *R. solanacearum*, as the focal strain. Previous work has shown that RAL5 efficiently colonizes the tomato rhizosphere and stem, and suppresses *R. solanacearum* primarily through resource competition rather than constitutive secretion of antimicrobial compounds [9, 27]. We therefore asked whether interaction with a partner could broaden the functional basis of RAL5-mediated biocontrol beyond resource competition by generating emergent antagonism. We first assessed the effects of monoculture supernatants from 89 tomato rhizosphere isolates on *R. solanacearum* growth and identified isolates with only weak inhibitory effects on their own, which allowed us to distinguish interaction-induced suppression from constitutive activity of a strongly inhibitory partner. A subset of such “weak-effect” bacterial isolates was then co-cultured with the RAL5 focal strain to test if pairwise co-culture supernatants showed a higher inhibitory effect compared to supernatants from either monoculture. We selected one resident community member pair (*R. pickettii* RAL5 and *Acinetobacter oleivorans* ACI4) with the strongest interaction-dependent pathogen inhibition for in-depth characterization of the interaction features, the transcriptional and metabolic responses underlying the emergent inhibition, and the disease-suppressive performance under greenhouse conditions. Our findings reveal that the competitive interaction between RAL5 and ACI4 resulted in enhanced pathogen suppression relative to either monoculture, coinciding with the accumulation of antimicrobial secondary metabolites and the release of intracellular contents from ACI4. The emergent antagonism also persisted in the tomato rhizosphere, providing improved disease control *in planta* relative to when each strain was applied alone. Together, this study provides a mechanistic framework for understanding how competition-mediated interactions within resident bacterial communities can trigger emergent antagonism and increased invasion resistance in rhizosphere microbiomes.

**Figure 1:**
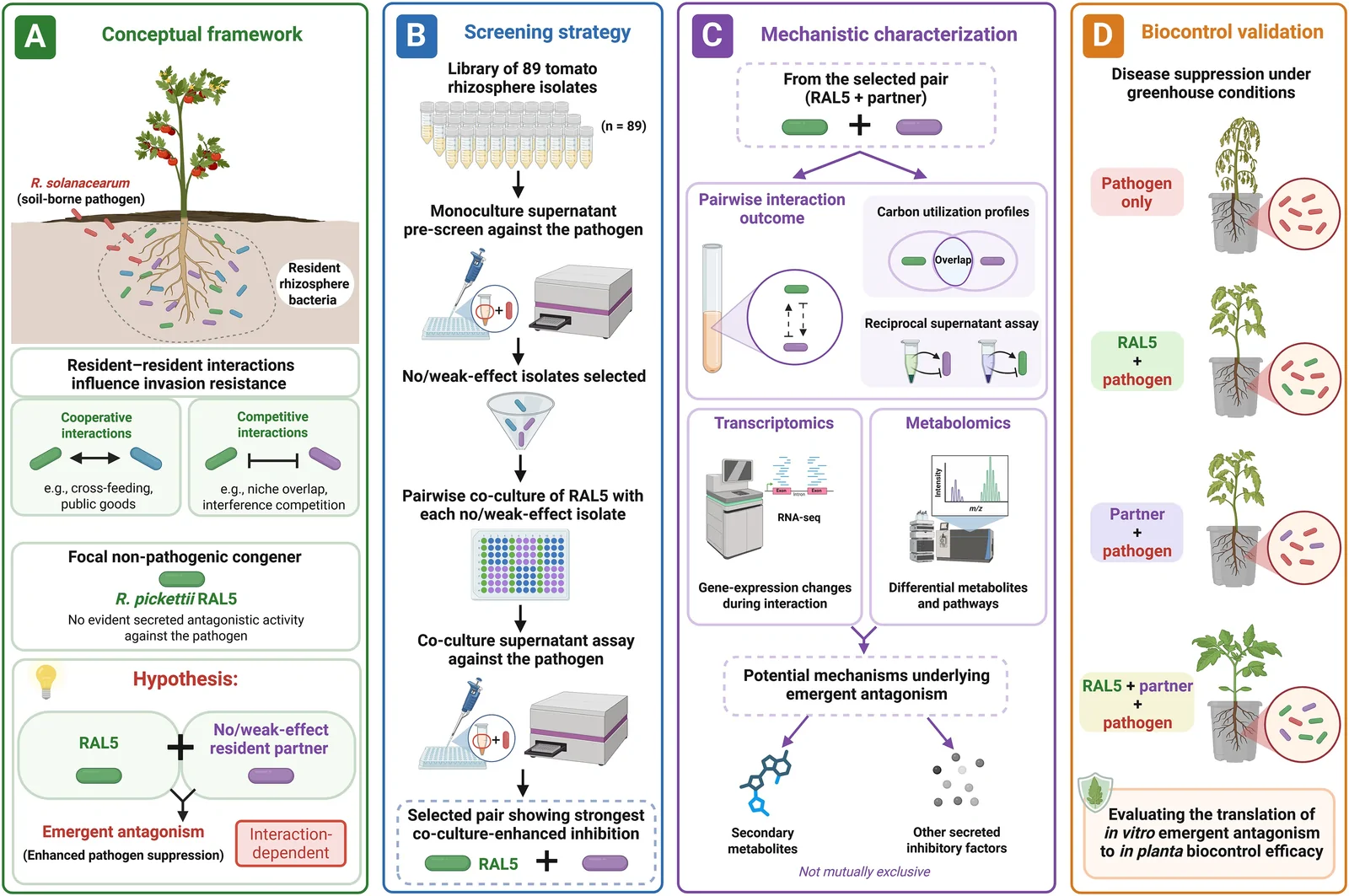
Conceptual overview of the interaction-centered strategy for identifying emergent antagonism in the tomato rhizosphere microbiome. (A) Resident–resident interactions in the rhizosphere can affect invasion resistance, motivating the hypothesis that interaction with another resident bacterium may add an antagonistic layer to the baseline biocontrol activity of *R. pickettii* RAL5 against *R. solanacearum*. (B) Rhizosphere isolates were pre-screened for weak monoculture effects and then tested in pairwise co-culture with RAL5 to identify combinations that enhanced pathogen inhibition. (C) The species pair with the highest inhibitory activity was selected and analyzed using interaction profiling, transcriptomics, and metabolomics to explore potential mechanisms underlying emergent antagonism. (D) Greenhouse assays were used to compare disease suppression by single-strain and consortium treatments, evaluating whether emergent antagonism observed in vitro translates into improved disease control. Created in BioRender. Yin, Y. (2026) https://BioRender.com/tfdyggj.

## Materials and Methods

### Bacterial strains and culture conditions

The phytopathogenic strain *R. solanacearum* QL-Rs1115 carrying plasmid pYC12-mCherry (hereafter Rs1115) was used as the model pathogen [30, 31]. *Ralstonia pickettii* QL-A6 (hereafter RAL5), a non-pathogenic congeneric strain previously shown to suppress *R. solanacearum* via resource competition [9, 27], was used as the fixed focal strain in the co-culture screen. A collection of 89 culturable bacterial isolates from the tomato rhizosphere in fields with recurrent bacterial wilt in Qilin Town, Nanjing, China (118°57′ E, 32°03′ N), was used to identify bacterial taxa that showed weak pathogen suppression on their own but potentially interacted with the RAL5 strain in co-cultures (Table S1; Fig. S1). These bacterial isolates span 22 families and 37 genera. To explore whether co-culture-enhanced pathogen suppression was specific to RAL5, or if it could also apply to other non-pathogenic but phylogenetically closely related *Ralstonia* spp. strains, four additional isolates, namely *R. mannitolilytica* QL-A2, *R. mannitolilytica* QL-A3, *Cupriavidus taiwanensis* QL-117 (formerly *Ralstonia* sp.), and *Cupriavidus* sp. QL-140 (formerly *Ralstonia* sp.), were included in the substitution assays [9]. *Escherichia coli* OP50 was also used as a non-target indicator strain to assess whether the inhibitory activity of RAL5–ACI4 co-culture supernatants extended beyond *R. solanacearum*.

Except for *E. coli* OP50, all bacterial species were streaked from frozen glycerol stocks onto nutrient agar (NA) plates (glucose 10.0 g/l, tryptone 5.0 g/l, yeast extract 0.5 g/l, beef extract 3.0 g/l, and agar 25.0 g/l; pH adjusted to 7.0) and incubated at 30°C. Single colonies were inoculated separately in nutrient broth (NB; same composition as NA but without agar) and cultured for ~16 h (late exponential phase) at 30°C with constant shaking at 170 rpm. *Escherichia coli* OP50 was streaked from frozen glycerol stocks onto lysogeny broth (LB) agar plates (tryptone 10.0 g/l, yeast extract 5 g/l, NaCl 10.0 g/l, and agar 15.0 g/l; pH adjusted to 7.0) and incubated at 37°C. Single OP50 colonies were inoculated in liquid LB (same composition without agar) and cultured overnight at 37°C with constant shaking at 170 rpm. Bacterial cells were harvested by centrifugation (3500× *g*, 5 min) and washed three times with sterile deionized water. The pellets were resuspended and adjusted to a concentration of approximately 10^8^ colony forming units (CFU) ml^−1^. Unless otherwise specified, cultures were diluted 100-fold into fresh medium from this starting density to initiate experiments.

### Screening for co-culture-enhanced pathogen suppression

To identify partner strains that could potentially enhance the suppressiveness of RAL5 to the pathogen in co-culture, we first evaluated the effects of monoculture supernatants derived from 89 rhizosphere isolates on Rs1115 growth. This was done to minimize the confounding influence of strongly inhibitory monoculture supernatants derived from partner strains to detect the co-culture-induced enhancement of pathogen inhibition. The strains whose monoculture supernatants produced pathogen inhibition values between −20% and +20% were classified as weak-effect strains and were retained for subsequent co-culture screening (Table S1; Fig. S1).

For co-culture screening, RAL5 was co-inoculated with each tested strain at a 1:1 ratio into 96-well microplates containing 200 μl NB medium per well, with a total initial inoculum of approximately 10^6^ CFU ml^−1^. Monocultures of RAL5 and each corresponding partner strain were prepared in parallel under the same conditions. After 48 h of incubation (30°C, 170 rpm), cell-free supernatants were collected by centrifugation (1650× *g*, 10 min) and filtration (0.22 μm). Inhibitory activity against Rs1115 was quantified by mixing 20 μl of each monoculture or co-culture supernatant with 2 μl of Rs1115 culture (OD_600_ = 0.50) in 178 μl of fresh 20% NB medium. Sterile 20% NB medium was used to replace the supernatant in the negative control. Pathogen growth was measured using optical density at 600 nm (OD_600_) after 24 h (30°C, 170 rpm). Inhibition efficiency was calculated as the percentage reduction in Rs1115 growth in the presence of each supernatant relative to the negative control. Enhanced inhibition was observed when co-culture supernatants exhibited stronger suppression than either of the two corresponding monoculture supernatants. To assess whether the partner strain effects were specific to RAL5, substitution assays were conducted where RAL5 was replaced by each of the four additional phylogenetically related non-pathogenic strains, with experimental details provided in Supplementary Methods.

### In vitro validation of pathogen suppression by the RAL5–ACI4 co-culture

Based on the co-culture supernatant screening, the RAL5–ACI4 pair was selected for in vitro validation due to its strong inhibitory effect on the pathogen. We then tested whether RAL5–ACI4 co-cultures enhanced pathogen suppression in direct cell contact with the Rs1115 pathogen relative to RAL5 and ACI4 monocultures. Briefly, 2 μl of bacterial inoculum (adjusted to approximately 10^8^ CFU ml^−1^) was mixed with 2 μl of Rs1115 (OD_600_ = 0.50) in 196 μl of fresh NB medium. For co-culture treatments, RAL5 and ACI4 were mixed at a 1:1 ratio before inoculation. Sterile water was used to replace bacterial inoculum in the pathogen-only control. Six independent culture replicates were established per treatment and incubated at 30°C with shaking. Rs1115 growth was monitored as mCherry fluorescence signal intensity (excitation 587 nm, emission 610 nm) with a SpectraMax M5 microplate reader (Molecular Devices, CA, USA). Cumulative pathogen growth was quantified as the area under the fluorescence–time curve (AUC). Inhibition was defined as a reduction in AUC relative to the pathogen-only control, with lower values corresponding to stronger suppression.

### Characterization of pairwise ecological interactions between RAL5 and ACI4

To quantify interaction outcomes during co-culture experiments, RAL5 and ACI4 were grown either in monoculture or in pairwise co-culture at an initial ratio of 1:1 in NB medium. At each 12-h interval over 72 h, three independent cultures per treatment were destructively harvested. Bacterial densities (CFU ml^−1^) were determined by plating serial dilutions on NA plates and distinguishing RAL5 and ACI4 colonies based on colony morphology. For each strain, the interaction index was calculated as the log_10_-transformed ratio of its cell density in co-culture relative to monoculture. Competition intensity was defined as the sum of the absolute values of the interaction indices of both partners [20]. To assess frequency-dependent effects on competitive outcomes, additional co-cultures were established at initial RAL5 to ACI4 ratios of 1:10, 1:100, 10:1, and 100:1, with the total initial cell density maintained at approximately 10^6^ CFU ml^−1^. Interaction outcomes were quantified after 48 h using the procedure described above. Additional assays characterizing resource-use overlap and supernatant-mediated effects between RAL5 and ACI4 are described in the Supplementary Methods.

### Transcriptomic sequencing and analysis of RAL5 and ACI4 gene expression in mono- and co-cultures

To investigate transcriptional changes in RAL5 and ACI4 gene expression, their mono- and 1:1 co-cultures were grown in NB medium at 30°C, and samples were collected at 24 h and 48 h from six biological replicates per treatment. Cell pellets were harvested by centrifugation, washed, flash-frozen, and stored at −80°C. Following RNA extraction and library preparation (detailed in Supplementary Methods), samples were sequenced on a NovaSeq 6000 System (Illumina) in paired-end mode (2× 150 bp) by Shanghai Biozeron Biotechnology Co., Ltd.

Raw reads were trimmed with Trimmomatic v0.36 [32] (SLIDINGWINDOW:4:15; MINLEN:75) and aligned to the annotated reference genomes of RAL5 and ACI4 using Rockhopper v2.0.3 [33] in orientation-aware mode (genome sources and annotation procedures are provided in Supplementary Methods). Differentially expressed genes were identified using edgeR [34, 35] with TMM normalization and thresholds of FDR < 0.05 and |log_2_FoldChange| > 1. RPKM values were calculated for expression summaries and visualization. Functional enrichment analyses were performed using KOBAS 3.0 for KEGG pathways [36], and pathways with hypergeometric test *P* < .05 were considered significantly enriched. To estimate the overall direction of differential expression within each enriched pathway, a Z-score [37] was calculated as:



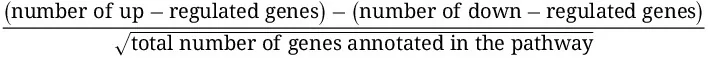



Global transcriptional variation among treatments was visualized using principal component analysis (PCA). Weighted gene co-expression network analysis was performed using the online WGCNA module implemented in ImageGP 2 [38, 39]. Transcriptomes from both 24 h and 48 h were analyzed jointly, with each sample retained as an individual observation, to identify co-expression modules associated with supernatant-mediated inhibition of Rs1115.

### Untargeted metabolomic profiling and analysis of RAL5 and ACI4 in mono- and co-cultures

To characterize extracellular metabolic changes associated with the interaction between RAL5 and ACI4, cell-free supernatants collected from all the treatments of the transcriptomic experiment at 48-h time point were used. Supernatants were frozen at −80°C and processed using an organic-solvent extraction protocol prior to untargeted LC–MS/MS profiling performed by Wuhan Metware Biotechnology Co., Ltd. Metabolite annotations were assigned to four confidence levels according to the platform-specific annotation workflow, and level 4 features were excluded from the downstream analysis. Detailed extraction procedures, annotation criteria, and raw data processing are provided in Supplementary Methods.

Global metabolic variation among treatments was assessed using unsupervised PCA analysis. To identify metabolites contributing to the separation between treatments, orthogonal partial least-squares discriminant analysis (OPLS-DA) was performed after log_2_ transformation and mean-centering of feature intensities. Variable importance in projection (VIP) values were extracted from the OPLS-DA model, and model robustness was evaluated using a 200-permutation test. For pairwise comparisons, univariate significance was assessed using Student’s *t-*test followed by Benjamini–Hochberg false discovery rate (FDR) correction. Differential metabolites were defined by VIP > 1, FDR < 0.05, and |log_2_FoldChange| > 1. Because the samples were derived from a rich medium, differential metabolites were manually curated before downstream interpretation. Putative metabolite identities were cross-referenced against public chemical databases, including PubChem and HMDB, and annotations corresponding to likely medium components, synthetic industrial chemicals, plasticizers, or other non-biological contaminants were excluded. After curation, metabolites were retained only if they were significantly enriched in the RAL5–ACI4 co-culture relative to both monocultures and were biologically plausible as bacterial metabolites.

### Testing the antibacterial activity of selected co-culture-enriched metabolite candidates

Commercial standards of 4-aminophenol (Thermo Scientific, USA), novobiocin, and tylosin B (Sigma-Aldrich, USA) were ordered and tested against Rs1115, RAL5, and ACI4 because these compounds were significantly enriched in the RAL5–ACI4 co-culture. Liquid susceptibility assays were used to assess the effects of all three compounds as follows. Bacterial cultures were inoculated into NB at approximately 10^6^ CFU ml^−1^ in 96-well plates and treated with either freshly prepared 4-aminophenol (200 μg/ml) or antibiotic standards (novobiocin or tylosin B, 100 μg/ml). Sterile water was used as the negative solvent control. After incubation at 30°C with shaking at 170 rpm for 24 h, bacterial growth in novobiocin and tylosin B treatments was quantified by OD_600_. Because 4-aminophenol underwent spontaneous oxidation and discoloration in liquid medium, which interfered with spectrophotometric measurements, its effect was evaluated by plating bacterial serial dilutions onto NA plates after 24 h exposure. The liquid assay was performed with four independent biological replicates. Agar spot assays were additionally used to compare novobiocin susceptibility of Rs1115, RAL5, and ACI4 across a concentration gradient, with details provided in the Supplementary Methods.

### Inhibitory activity of ACI4 intracellular lysate on pathogen growth

Because RAL5 could outcompete ACI4, we assessed if intracellular contents released upon ACI4 lysis could also contribute to bacterial growth suppression by preparing intracellular lysate from ACI4 monocultures grown for 48 h and testing its effect on RAL5 and Rs1115 growth in liquid assays. ACI4 cultures were centrifuged at 3500× *g* for 5 min at 4°C, and the remaining cell pellet was washed twice with sterile PBS to remove residual extracellular metabolites. Cell pellets were then resuspended in PBS and lysed by four freeze–thaw cycles (alternating between liquid nitrogen for 5 min and a 37°C water bath for 5 min). Lysates were clarified by centrifugation at 9600× *g* for 5 min at 4°C and filtered through a 0.22 μm membrane before use. The lysate was then tested against RAL5 and Rs1115 under the same liquid inhibition conditions used in the supernatant assays. Briefly, bacterial suspensions were supplemented with 10% (v/v) intracellular lysate in 20% NB medium, with PBS used as the negative control. After 24 h of incubation at 30°C with shaking at 170 rpm, bacterial growth was quantified by OD_600_ and inhibition was calculated relative to the negative control. For a secretion control, the extracellular supernatant from the same ACI4 cultures grown for 48 h was tested under the same conditions. All experiments were performed in three independent biological replicates.

### Greenhouse evaluation of RAL5–ACI4 consortium on bacterial wilt suppression and rhizosphere colonization dynamics

To evaluate whether RAL5, ACI4, and their 1:1 consortium could suppress bacterial wilt under rhizosphere conditions, a greenhouse pot experiment was performed using tomato seedlings (*Solanum lycopersicum* cv. Moneymaker). Seedlings at the three-leaf stage were transplanted into sterilized potting soil (Biolan Peat-Free Garden Soil, Finland; 200 g per pot) that had been autoclaved twice with a 5-day interval between sterilization cycles. Plants were maintained under greenhouse conditions with temperature ranging from 28°C to 32°C. After 7 days of acclimation, plants were inoculated with RAL5, ACI4, or their 1:1 consortium by root drenching using 20 ml of bacterial suspension prepared in sterile water, with a final density of approximately 10^7^ CFU g^−1^ soil. Seven days later, plants were inoculated with Rs1115, which was prepared similarly to the resident bacterial inoculants but at a lower pathogen density of approximately 10^6^ CFU g^−1^ soil. Two controls were included: a non-inoculated control (sterile water only) and a pathogen-only control (no prior inoculation with resident bacteria).

Each treatment consisted of three independent replicate trays, with each tray initially containing eight plants. After 2 and 7 days of resident-strain inoculation, one plant per tray was randomly selected and destructively sampled to quantify temporal changes in RAL5 and ACI4 rhizosphere abundances. To achieve this, rhizosphere soil tightly adhered to the plant roots was collected for quantitative PCR (qPCR) analysis. Immediately after the Day 7 sampling, the remaining six plants per tray were challenged with the pathogen and used for disease assessment. Trays were rearranged randomly every two days and watered regularly. Following pathogen inoculation, disease progression was monitored daily until no newly wilted plants were observed. The experiment was terminated 35 days after the initial inoculation with RAL5, ACI4, or their consortium. Disease severity was scored on a 0–4 wilting scale, and the disease index was calculated separately for each replicate tray based on the number of wilted plants at each severity level [40]. Following final disease scoring, one additional plant per tray was destructively sampled for rhizosphere soil collection. Bacterial abundance was quantified by qPCR. The pathogen Rs1115 was detected using the established *fliC* gene primers [41]. RAL5 and ACI4 were quantified using strain-specific primers targeting unique single-copy marker genes, which were identified by comparative genomic analysis [42] (Table S2). Detailed rhizosphere soil collection, DNA extraction, primer design, standard curve generation, and qPCR conditions are provided in Supplementary Methods.

### Statistical analysis

Statistical analyses were performed in R (v4.4.2) [43]. Bacterial abundance data were log_10_-transformed, and disease index percentages were arcsine square root transformed when required to attain normality and homogeneity of variance. Two-group comparisons were performed using Welch’s *t-*test. Multi-group comparisons were analyzed using one-way ANOVA followed by Tukey’s HSD or Dunnett’s test, Welch’s one-way ANOVA followed by Games–Howell tests, or Kruskal–Wallis test followed by Dunn’s test, as appropriate. Rs1115 mCherry fluorescence AUC was calculated by trapezoidal integration over 0–72 h. Non-additivity of the RAL5 and ACI4 effects on Rs1115 mCherry fluorescence was assessed using separate 2 × 2 factorial linear models for natural-log-transformed fluorescence AUC and fluorescence at 72 h. RAL5 presence, ACI4 presence, and their interaction were included as fixed effects. The exponentiated RAL5 × ACI4 interaction coefficient was interpreted as the observed-to-expected ratio under multiplicative independence. For the in vitro pairwise interaction assay, population densities across sampling times were analyzed by two-way ANOVA, with sampling time, culture condition (monoculture versus co-culture), and their interaction included as categorical fixed effects (no population was measured repeatedly). A linear model was used to investigate the relationship between competition intensity and supernatant-mediated pathogen inhibition. A whole-genome phylogenomic tree was constructed from Mash distances using Mashtree (v0.37) and visualized with iTOL v7 [44]. Global differences in transcriptomic and metabolomic profiles among treatments were evaluated using permutational multivariate analysis of variance (PERMANOVA; 999 permutations) based on Euclidean distances, using the adonis2 function in the vegan package v2.7-5 [45]. Statistical significance was defined as *P* < .05.

## Results

### *Acinetobacter* strain ACI4 triggers strong emergent pathogen suppression in co-culture with RAL5

Previous research has demonstrated that *R. pickettii* RAL5 can suppress the phytopathogenic *R. solanacearum* Rs1115 primarily through resource competition instead of secretion of antimicrobial compounds [9, 27]. We therefore screened resident rhizosphere isolates for their ability to enhance secretion-mediated pathogen suppression when co-cultured with RAL5. Because strongly inhibitory monoculture supernatants would obscure the detection of interaction-dependent enhancement, we first screened monoculture supernatants from 89 candidate resident isolates and selected 51 weak-effect strains (on their own) for subsequent co-culture screening (Table S1). For most resident strains, co-culturing with RAL5 did not significantly alter pathogen inhibition relative to their corresponding monocultures, whereas 6 out of 51 strains showed significantly enhanced inhibitory activity in co-culture and another 6 out of 51 showed reduced pathogen inhibition (*P* < .05; Fig. 2A; Table S3). Among the six strains that enhanced pathogen suppression, four belonged to the genus *Acinetobacter*, including strains ACI4, ACI5, ACI7, and ACI11. *Acinetobacter oleivorans* ACI4 showed the strongest increase in inhibitory activity when co-cultured with RAL5, reducing pathogen growth by 83.0% (RAL5 monoculture: 7.4%; ACI4 monoculture: 5.0%; Fig. 2A; Table S1). To assess whether the ACI4-mediated increase in pathogen suppression was specific to the RAL5 strain, we substituted RAL5 with four additional non-pathogenic but closely related *Ralstonia* spp. strains and repeated the assay using ACI4 as the partner. Co-culturing with ACI4 increased pathogen inhibition across all pairings, but the RAL5–ACI4 pair exhibited the strongest inhibitory activity (*F*_4,10_ = 19.8, *P* < .001; Fig. S2). Therefore, we chose the RAL5–ACI4 pair for subsequent analyses to better understand the underlying mechanisms of observed emergent antagonism.

**Figure 2:**
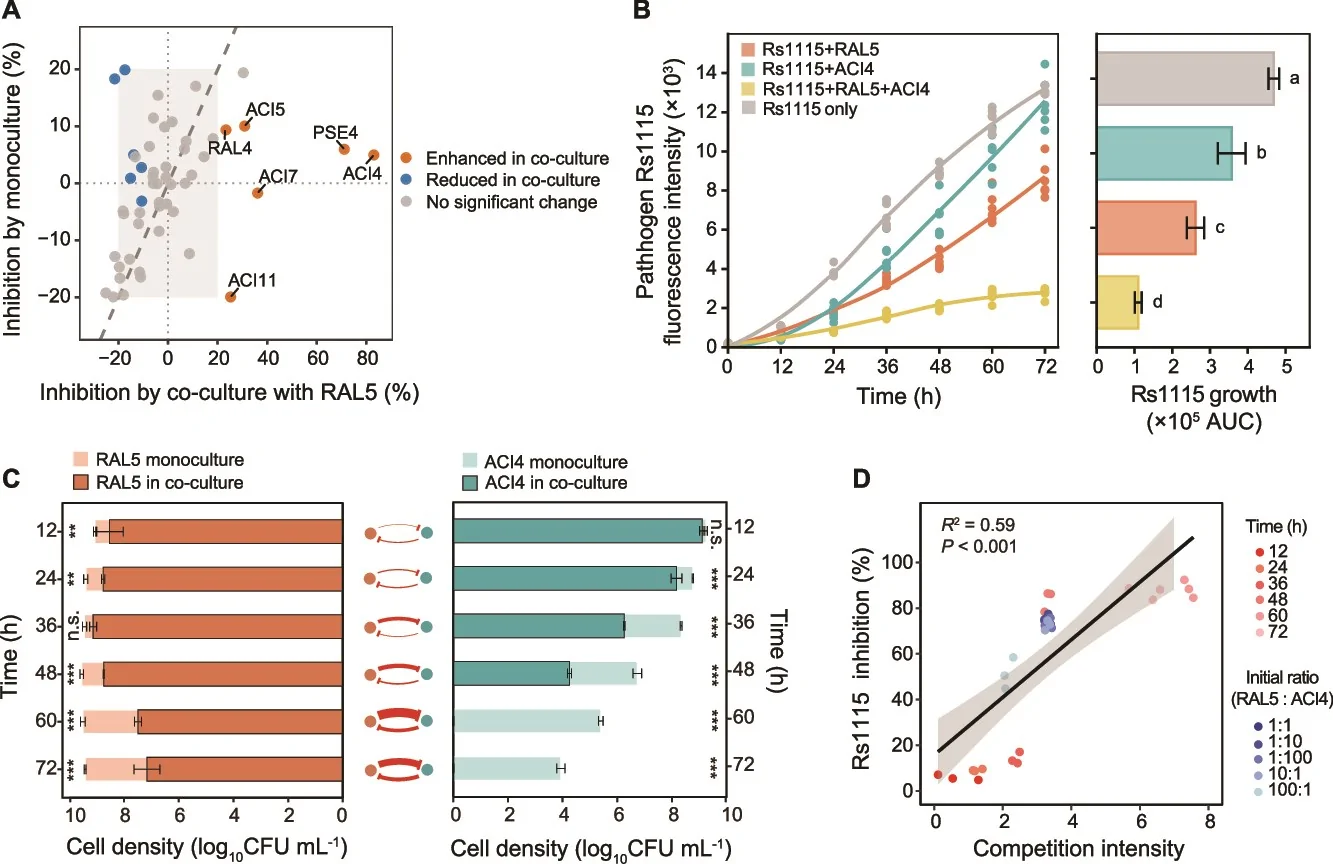
Emergent pathogen suppression and interspecific interactions in RAL5 and ACI4 mono- and co-cultures. (A) Screening of 51 weak-effect rhizosphere isolates for changes in supernatant-mediated inhibition of *R. solanacearum* Rs1115 when co-cultured with RAL5. Each point represents one isolate. The dashed diagonal line indicates *y* = *x*, representing equal inhibitory effects of monoculture and co-culture supernatants. Point colors indicate significantly enhanced (red), significantly reduced (blue), or non-significant changes (gray) in inhibition by co-culture supernatants relative to the corresponding monoculture supernatants. Labels indicate enhanced hits with mean co-culture inhibition >20%. (B) Pathogen suppression measured directly in bacterial co-cultures. Left: temporal dynamics of Rs1115 mCherry fluorescence in the presence of RAL5, ACI4, or the RAL5–ACI4 co-culture, compared to the pathogen-only control. Right: cumulative Rs1115 mCherry fluorescence quantified as the area under the curve (AUC). (C) Population dynamics and interspecific interaction intensity of RAL5 and ACI4. Cell densities of RAL5 (orange) and ACI4 (teal) in monoculture (lighter bars) and co-culture (darker bars) are shown over 72 h. The central schematics summarize the reciprocal competition between the two strains (the wider the line, the stronger the competition). (D) Linear regression with 95% confidence interval (shaded area) between competition intensity (calculated as the sum of the absolute interaction indices of RAL5 and ACI4) and pathogen inhibition efficacy (supernatant-mediated suppression of Rs1115) across different co-culture ratios and time points. In panel (A), statistical significance was determined by Welch’s *t-*tests with Benjamini–Hochberg correction, comparing the inhibitory effects of supernatants produced by each strain in monoculture versus in co-culture with RAL5 (*P* < .05). In panel (B), different letters indicate significant differences among treatments based on Welch’s one-way ANOVA followed by Games–Howell post hoc comparisons (*P* < .05). In panel (C), asterisks indicate significant differences in cell density between monoculture and co-culture at each time point (two-way ANOVA followed by post hoc comparisons; ^*^P < .05, ^**^P < .01, ^***^P < .001, n.s. not significant). Data with error bars represent mean ± SD. Sample sizes are provided in the Methods.

### Competition between RAL5 and ACI4 activates sustained pathogen suppression

Because the initial screening was based on cell-free supernatants, we next examined whether pairing RAL5 with ACI4 also suppressed Rs1115 growth when the two resident strains and the pathogen were grown together in co-culture. In pairwise co-cultures with Rs1115, both RAL5 and ACI4 reduced cumulative Rs1115 fluorescence relative to the Rs1115-only control (both *P* ≤ .001). The three-strain co-culture yielded the lowest mean AUC, which was significantly lower than those of the Rs1115-only control and both RAL5 and ACI4 single-strain treatments (all *P* < .001; Fig. 2B). Factorial analysis showed that Rs1115 mCherry fluorescence in the three-strain co-culture was 44.5% lower for AUC and 65.9% lower at 72 h than expected if RAL5 and ACI4 acted independently (both *P* < .001; Table S4), indicating a significant non-additive enhancement of pathogen suppression. After 48 h, the difference in mean Rs1115 fluorescence between the three-strain co-culture and the other treatments progressively increased (Fig. 2B). This temporal pattern coincided with the onset of inhibitory activity observed in RAL5–ACI4 co-culture supernatants: supernatants collected from early co-culture time points (12–36 h) exerted little effect on Rs1115, whereas the pathogen inhibition became evident only in supernatants harvested after 48 h of co-culturing (48 h: *F*_2,6_ = 395.0, *P* < .001; Fig. S3). To test whether this emergent antagonism extended beyond *R. solanacearum*, we assessed the co-culture supernatant effects against *E. coli* OP50. Relative to both monoculture controls, the RAL5–ACI4 co-culture supernatant inhibited the growth of OP50 more strongly (*P* < .001; Fig. S4), indicating that the observed antagonism was not restricted to Rs1115.

To examine whether resource competition could partly explain the stronger suppression of Rs1115 in the three-strain co-culture, we compared carbon-use profiles among RAL5, ACI4, and Rs1115 across 48 common rhizosphere carbon sources. The combined resource-use profile of RAL5 and ACI4 showed slightly greater overlap with Rs1115 (overlap index = 0.70) than either resident strain alone (RAL5–Rs1115: 0.63; ACI4–Rs1115: 0.68). However, the highest overlap was observed between RAL5 and ACI4 (0.77; Fig. S5; Table S5). We next explored competition between the two resident species and found that both RAL5 and ACI4 reached lower cell densities in co-cultures compared to their respective monocultures (two-way ANOVA, treatment effects; RAL5: *F*_1,24_ = 238.4, *P* < .001; ACI4: *F*_1,24_ = 5527.7, *P* < .001; Fig. 2C). The strength of competition increased over time (two-way ANOVA, treatment × time interaction; RAL5: *F*_5,24_ = 22.8, *P* < .001; ACI4: *F*_5,24_ = 620.5, *P* < .001; Fig. 2C), with ACI4 declining more strongly and falling below the detection limit by 60 h of co-culture. Consistent with this decline, RAL5–ACI4 co-culture supernatants collected at 48 h strongly inhibited ACI4 but had little effect on RAL5 (Fig. S6A). Because supernatants from RAL5 or ACI4 monocultures did not significantly inhibit the other strain (*P* = .138 and .134; Fig. S6B), the strong inhibition of ACI4 appeared to depend on the RAL5–ACI4 co-culture interaction. Although the substantial overlap in resource-use profiles indicates the potential for resource competition between RAL5 and ACI4, the relatively modest effects of co-culture on strains’ peak population densities provide limited support for strong competition over limiting resources. In contrast, the pronounced late-stage decline of ACI4 and the inhibitory activity of 48-h co-culture supernatants point to a prominent role of co-culture-dependent secretion in shaping the asymmetric competitive outcome and emergent antagonism against Rs1115.

Given the extinction of ACI4 in pairwise co-cultures, we next tested whether this competitive outcome and emergent antagonism were sensitive to the initial inoculation ratio of RAL5 and ACI4. Across most ratios ranging from 1:100 to 100:1, co-culture supernatants retained strong inhibitory activity against Rs1115 (Fig. S7A). However, when ACI4 was introduced at a very low initial ratio (RAL5:ACI4 = 100:1), the resulting co-culture supernatants showed a clear decline in pathogen inhibition (*F*_4,10_ = 24.7, *P* < .001; Fig. S7A), which was also accompanied by a significant weakening of competitive suppression between the two strains (ACI4 to RAL5: *F*_4,10_ = 26.4, *P* < .001; RAL5 to ACI4: *F*_4,10_ = 89.7, *P* < .001; Fig. S7B). Linear regression analysis further confirmed that competition intensity between RAL5 and ACI4 was positively associated with the pathogen suppression (*R*^2^ = 0.59, *P* < .001; Fig. 2D). Overall, these results indicate that activation of pathogen suppression required a sufficient initial abundance of ACI4 and was closely linked to the strength of competition between the two resident strains.

### Interspecific competition leads to asymmetric transcriptional reprogramming in RAL5–ACI4 co-culture

To understand the underlying mechanisms of emergent antagonism observed in RAL5–ACI4 co-culture, we performed comparative transcriptomic profiling of RAL5 and ACI4 in mono- and co-cultures after 48 h of incubation, corresponding to the onset of strong supernatant-mediated pathogen suppression (Fig. S3). RNA sequencing generated approximately 100 million clean reads per treatment across six biological replicates. In monoculture libraries, an average of 74% of reads were mapped to the RAL5 genome and 53% mapped to the ACI4 genome. In co-culture libraries, 40% and 34% of reads were mapped to the RAL5 and ACI4 genomes, respectively. After filtering out genes with low expression levels across all samples (RPKM <1 in all samples), 4947 RAL5 genes and 3650 ACI4 genes were retained for the downstream analysis. PCA revealed transcriptional reprogramming in both species under co-culture versus monoculture conditions, but the response was substantially stronger in RAL5 than in ACI4 (PERMANOVA, RAL5: *F*_1,10_ = 39.6, *R*^2^ = 0.80, *P* = .005; ACI4: *F*_1,10_ = 5.0, *R*^2^ = 0.33, *P* = .005; Fig. 3A). This asymmetry was also apparent in DEG counts, with 584 DEGs detected in RAL5 (312 upregulated and 272 downregulated) but only 37 DEGs in ACI4 (30 upregulated and 7 downregulated; Fig. 3B).

**Figure 3:**
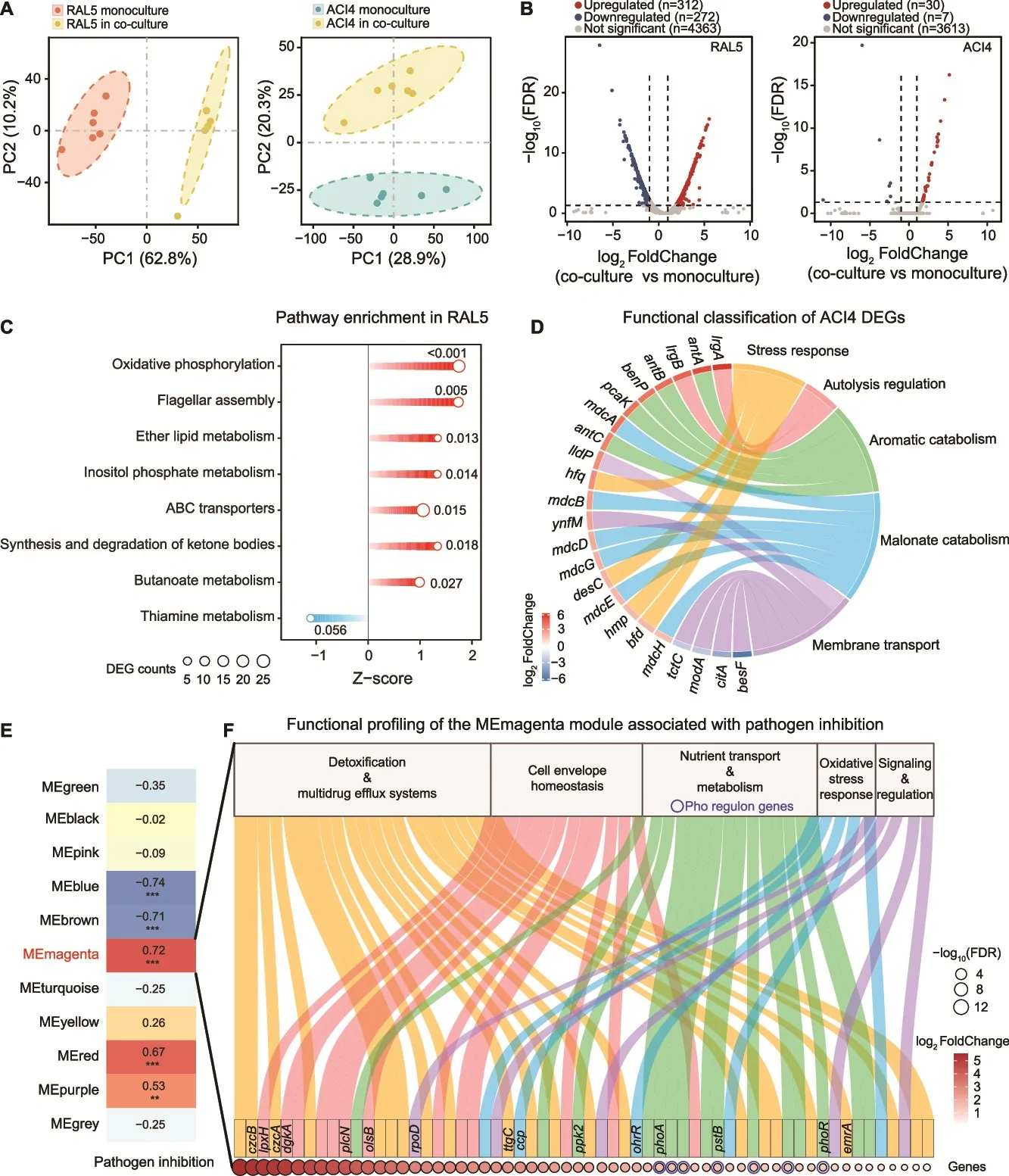
Transcriptional changes in the RAL5–ACI4 co-culture compared to monocultures of each strain. (A) PCA reveals distinct expression profiles for RAL5 (left) and ACI4 (right) in mono- and co-cultures. (B) Volcano plots display differentially expressed genes (DEGs) of RAL5 (left) and ACI4 (right) in co-culture compared to their respective monocultures. Significantly upregulated and downregulated genes are shown (FDR < 0.05, |log_2_FoldChange| > 1). (C) KEGG pathway enrichment analysis of DEGs in RAL5. Bubble size scales with gene count, and the Z-score indicates the regulation direction relative to the monocultures, with positive values indicating upregulation and negative values indicating downregulation of genes in co-culture. (D) Chord diagram mapping of key ACI4 DEGs according to their functional classification. (E) WGCNA showing RAL5 gene modules correlated with pathogen inhibition. The heatmap displays the correlation coefficients and significance (^**^P < .01, ^***^P < .001) between gene modules and Rs1115 growth suppression. (F) Functional mapping of the MEmagenta module, which showed the strongest positive correlation with pathogen inhibition. Genes are grouped by functional categories. The circled genes belong to the Pho regulon, highlighting a potential role for phosphate starvation response.

The RAL5 KEGG enrichment indicated coordinated activation of energy metabolism, motility, membrane-related processes, and transport functions, including oxidative phosphorylation (*P* < .001), flagellar assembly (*P* = .005), ABC transporters (*P* = .015), and pathways related to ether lipid (*P* = .013) and inositol phosphate metabolism (*P* = .014; Fig. 3C). Components of Type II, Type III, and Type VI secretion systems also showed a general trend toward transcriptional induction, which was not observed in ACI4 (Fig. S8). Secondary metabolism analysis further indicated activation of biosynthetic potential in RAL5 under co-culture conditions, and pathways related to secondary metabolite biosynthesis and terpenoid backbone biosynthesis were consistently upregulated (Fig. S8). Genome mining using antiSMASH identified multiple BGCs in the RAL5 genome (Table S6). Among these, a ribosomally synthesized and post-translationally modified peptide (RiPP)-like cluster showed the strongest induction, with key biosynthetic genes reaching expression levels of log_2_FoldChange ≈ 4 (FDR < 0.001; Table S6; Fig. S9). Additionally, arylpolyene clusters, typically associated with oxidative stress protection, were also induced. In contrast, no comparable activation of BGCs was detected in ACI4 (Table S6; Fig. S9).

In ACI4, the limited transcriptional response was mainly associated with catabolism and stress adaptation (Fig. 3D). Consistent with the observed capacity of ACI4 to utilize malonate (Fig. S5A), genes involved in malonate decarboxylation (the *mdc* operon) were significantly induced under co-culture conditions. Genes encoding the anthranilate dioxygenase complex (the *ant* operon) were also upregulated. In agreement with this pattern, anthranilic acid was abundant in RAL5 monocultures but significantly reduced in the RAL5–ACI4 co-culture (*P* < .001; Fig. S10). The *lrgAB* operon, which modulates murein hydrolase activity and autolysis [46], was induced together with several stress-related genes, including *hfq* and *hmp*. Together, these changes suggest that ACI4 responded in co-culture primarily through metabolic adjustments and stress-associated maintenance of cell envelope integrity.

Because these results suggested that the emergent antagonism was accompanied by RAL5-centered transcriptional reprogramming, we performed a weighted gene co-expression network analysis (WGCNA) using the RAL5 transcriptome to identify co-expression modules associated with pathogen suppression. Three modules were positively correlated with the pathogen inhibition. Among them, MEmagenta showed the strongest association (Pearson’s *r* = 0.72, *P* < .001; Fig. 3E) and genes within this module were enriched in detoxification and multidrug efflux functions, including *czcAB, ttgC*, and *emrA* (Fig. 3F). These genes were also co-expressed with enzymes involved in cell envelope maintenance and membrane remodeling (*lpxH, dgkA, plcN*, and *olsB*), together with components of the Pho regulon (*pstABC, phoA, ppk2*, and *phoR*; Fig. 3F; Table S7). The other two positively correlated modules, MEred and MEpurple, captured complementary defense mechanisms. MEred was characterized by genes involved in protein quality control (*groEL, hsp20*, and *ftsH*) and reactive oxygen species (ROS) scavenging (*ahpC* and *ahpD*; Table S7). MEpurple contained a more heterogeneous set of accessory genes, including membrane-associated proteins, MFS-type transporters, and several regulatory genes (Table S7). Together, these co-expression patterns reveal that emergent antagonism coincided with a broad defense response in RAL5. Rather than pointing to a discrete antimicrobial biosynthetic module, these results suggest that RAL5 mounted a producer-side protective program that may sustain suppressive output while limiting self-damage.

### Co-culturing RAL5 and ACI4 reshapes the chemical environment and reveals candidate metabolites underlying pathogen suppression

To identify metabolites enriched in RAL5–ACI4 co-culture that could have mediated emergent pathogen suppression, we performed untargeted LC–MS/MS profiling from cell-free supernatants of RAL5 and ACI4 mono- and co-cultures after 48 h of growth. Untargeted profiling initially detected 7614 metabolic features across positive and negative ion modes. After annotation and filtering, 4606 annotated metabolites (covering levels 1–3) were retained for downstream analysis. PCA showed that the co-culture metabolome was clearly separated from both monoculture metabolomes that also differed from each other (PERMANOVA: *F*_2,15_ = 9.9, *R*^2^ = 0.57, *P* = .001; Fig. 4A), indicating that co-culturing RAL5 and ACI4 substantially reshaped the chemical environment.

**Figure 4:**
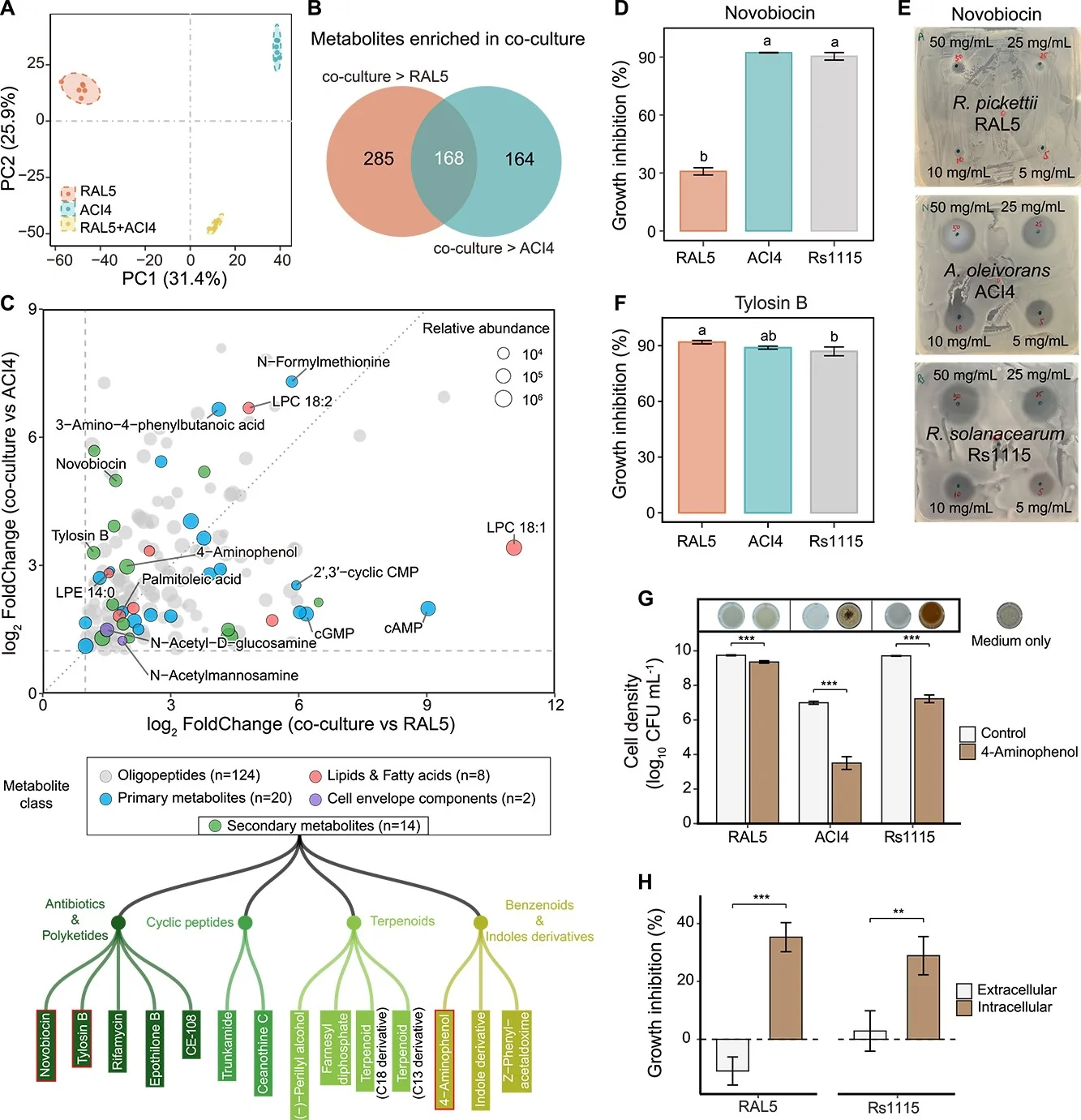
Chemical shifts in the RAL5–ACI4 co-culture reveal candidate suppressive metabolites and lysis-associated inhibitory effects. (A) PCA of untargeted metabolomic profiles for RAL5 monoculture, ACI4 monoculture, and the RAL5–ACI4 co-culture. The co-culture exhibited a distinct metabolomic profile relative to both monocultures. (B) Venn diagram showing metabolites significantly enriched in the RAL5–ACI4 co-culture compared with either RAL5 or ACI4 monoculture. The intersection comprises 168 metabolites that were consistently enriched in the co-culture relative to both monocultures and were used for downstream annotation and candidate metabolite selection. (C) Metabolites significantly enriched in the co-culture relative to both RAL5 and ACI4 monocultures. Bubble size scales with mean relative abundance in the RAL5–ACI4 co-culture, and colors denote manually grouped metabolite categories. Key metabolites are labeled by name. The bottom schematic summarizes the classification of annotated secondary metabolite candidates. (D) Growth inhibition of RAL5, ACI4, and Rs1115 following exposure to 100 μg/ml novobiocin. (E) Representative images of agar spot assays showing the susceptibility of RAL5, ACI4, and Rs1115 to novobiocin across a concentration gradient. (F) Growth inhibition of RAL5, ACI4, and Rs1115 following exposure to 100 μg/ml tylosin B. (G) Antimicrobial activity of 4-aminophenol. Top: representative growth phenotypes in liquid medium treated with water control or 200 μg/ml 4-aminophenol. The medium-only control (top right) indicates baseline auto-oxidation of 4-aminophenol. Bottom: viability counts of RAL5, ACI4, and Rs1115 treated with 4-aminophenol or water control. (H) Growth inhibition of RAL5 and Rs1115 mediated by ACI4 extracellular supernatants and intracellular lysates. In panels (D, F), different letters indicate statistically significant differences among strains based on one-way ANOVA followed by Tukey’s HSD test (*P* < .05). In panels (G, H), statistical significance was determined by Welch’s t-tests (^*^P < .05, ^**^P < .01, ^***^P < .001). All error bars represent mean ± SD.

Differential metabolite analysis identified 168 metabolites significantly enriched in the co-culture relative to both monocultures (Fig. 4B). The enriched metabolite profile was dominated by oligopeptides but also contained a smaller set of putative bioactive secondary metabolite candidates annotated as antibiotic-like, polyketide-like, cyclic peptide-like, terpenoid-related, and benzenoid compounds (Fig. 4C; Table S8). To assess whether these candidates could contribute to pathogen suppression, we selected and tested commercially available standards corresponding to representative annotated features (novobiocin, tylosin B, and 4-aminophenol). In the RAL5–ACI4 co-culture, the corresponding mean relative abundances were 16 648 for novobiocin, 20 894 for tylosin B, and 135 264 for 4-aminophenol (Table S8). Among the antibiotic-like annotations, novobiocin and tylosin B standards inhibited both ACI4 and the Rs1115 pathogen. RAL5 displayed substantially greater resistance to novobiocin (*F*_2,9_ = 2102.9, *P* < .001; Fig. 4D, E), whereas tylosin B suppressed all tested strains, including RAL5 (*F*_2,9_ = 10.4, *P* = .005; Fig. 4F). We also validated the antimicrobial activity of the benzenoid 4-aminophenol (C_6_H_7_NO), which had high identification confidence (Level 1; Table S8) and potential redox activity. We found that exposure to 200 μg/ml 4-aminophenol significantly reduced the viable cell counts of ACI4 and Rs1115, whereas RAL5 exhibited a smaller but still detectable reduction under the same conditions (RAL5: *t*_4.43_ = 11.47; ACI4: *t*_3.41_ = 17.42; Rs1115: *t*_3.05_ = 22.30; all *P* < .001; Fig. 4G). Consistent with this differential sensitivity, pronounced oxidative darkening was observed in cultures of ACI4 and Rs1115, whereas RAL5 cultures showed weaker discoloration (Fig. 4G), suggesting that RAL5 was less susceptible to 4-aminophenol-associated oxidative stress. This phenotype corresponded to the RAL5 co-culture transcriptome, in which arylpolyene biosynthetic genes and genes involved in multidrug efflux and cell envelope maintenance were upregulated (Fig. S9; Fig. 3F).

Given the rapid extinction of ACI4 post 48 h in the RAL5–ACI4 pairwise interaction assay (Fig. 2C), we hypothesized that cellular contents released from lysed ACI4 cells might also contribute to pathogen suppression. In support of this, the co-culture metabolome was enriched in membrane- and envelope-associated metabolites, including lysophospholipids (LPC 18:1, LPC 18:2, and LPE 14:0), free fatty acids (palmitoleic acid), and cell wall-related amino sugars (N-acetyl-D-glucosamine and N-acetylmannosamine; Fig. 4C). Moreover, nucleotide-derived metabolites, including RNA degradation intermediates such as 2′,3′-cyclic CMP, were also enriched in the co-culture supernatant (Fig. 4C), indicative of increased turnover of intracellular material following cell lysis. To test this experimentally, we prepared intracellular lysates from ACI4 monocultures and evaluated their antimicrobial activity against RAL5 and Rs1115. Compared to the non-inhibitory extracellular ACI4 fraction, the intracellular ACI4 lysates caused significant growth inhibition of both RAL5 and Rs1115 (RAL5: *t*_4.00_ = −11.47, *P* < .001; Rs1115: *t*_3.99_ = −4.69, *P* = .009; Fig. 4H). These results suggest that ACI4 cell lysis could also partly explain the increased suppressiveness of RAL5–ACI4 co-culture supernatants, along with the interaction-associated accumulation of antimicrobial compounds.

### RAL5–ACI4 consortium enhances bacterial wilt suppression in the tomato rhizosphere

To determine whether the emergent suppressiveness between RAL5 and ACI4 observed in vitro also holds under rhizosphere conditions, we evaluated the biocontrol performance of RAL5, ACI4, and their co-culture consortium in a tomato greenhouse experiment using sterilized soil inoculated with *R. solanacearum* Rs1115 strain. The ACI4 single-strain treatment did not reduce the disease index compared to the pathogen-only control (*P* = .806), whereas the RAL5 single-strain treatment showed a moderate but non-significant reduction (22%, *P* = .401; Fig. 5A). Consistent with the strongest in vitro pathogen suppression observed in the presence of both resident strains, the RAL5–ACI4 consortium significantly lowered the disease index by 44% (*P* = .023; Fig. 5A). Improved plant protection was accompanied by reduced pathogen densities in the rhizosphere. Co-inoculation with RAL5 and ACI4 yielded the lowest mean Rs1115 density and was the only treatment that differed significantly from the pathogen-only control (*P* = .043; Fig. 5B).

**Figure 5:**
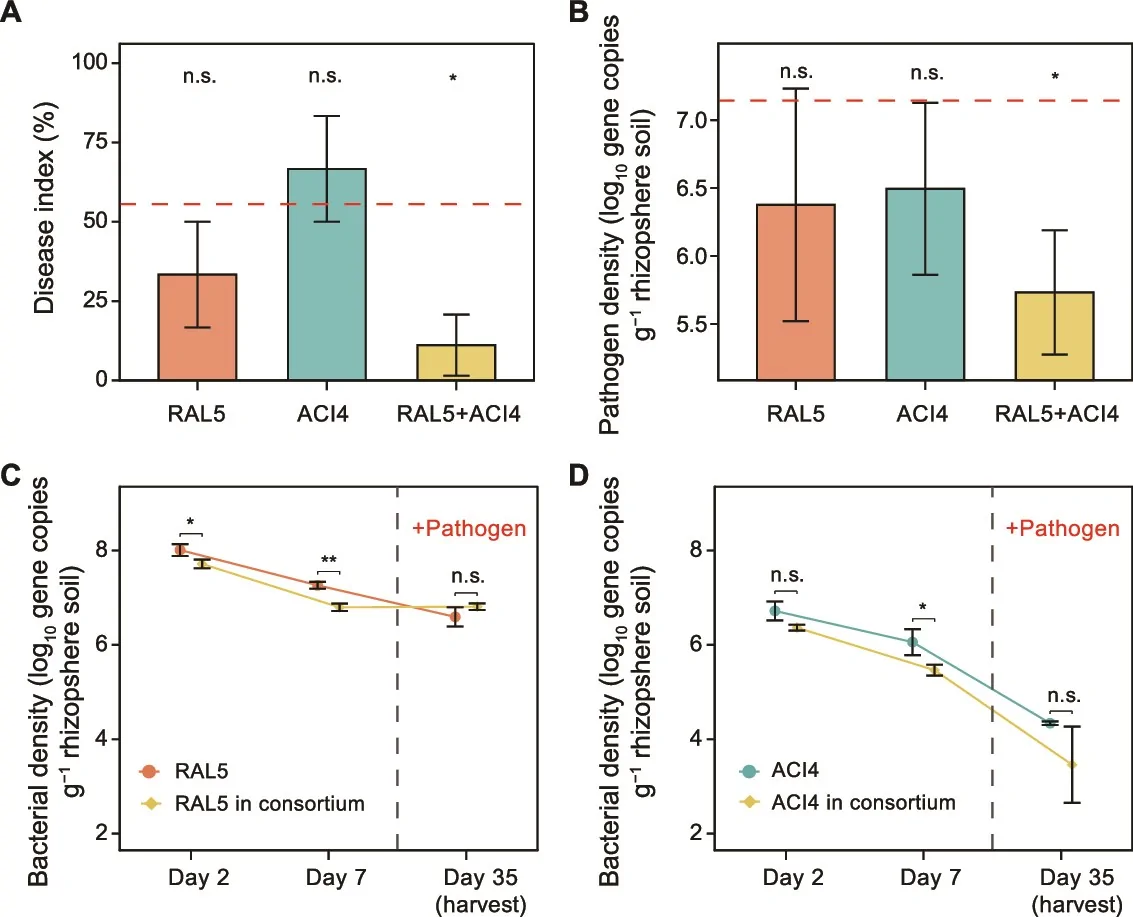
Biocontrol efficacy and rhizosphere colonization dynamics of the RAL5–ACI4 consortium. (A) Disease index of tomato bacterial wilt at harvest. (B) Abundance of pathogen Rs1115 in rhizosphere soil at harvest. Rhizosphere colonization dynamics of RAL5 (C) and ACI4 (D) under single-strain treatment versus consortium treatment (RAL5–ACI4 co-inoculation). Sampling time points capture early colonization (Day 2), stable colonization prior to pathogen inoculation (Day 7), and persistence after pathogen inoculation at harvest (Day 35). In panels (A, B), the horizontal dashed lines represent the mean values of the pathogen-only control. Statistical comparisons against the pathogen-only control were performed using Dunnett’s test on transformed data. In panels (C, D), significant differences between single-strain and consortium treatment at each time point were determined by Welch’s t-test. In all panels, ^*^P < .05, ^**^P < .01, ^***^P < .001 and n.s. indicates not significant. Error bars show mean ± SD.

To investigate if the competitive exclusion of ACI4 observed in vitro also took place in the rhizosphere, we quantified rhizosphere population dynamics of RAL5 and ACI4 using qPCR (Fig. 5C and D). Before the Rs1115 inoculation, RAL5 density was significantly lower under co-inoculation than in the RAL5 single-strain treatment at Day 2 (*t*_3.65_ = 3.28, *P* = .035) and Day 7 (*t*_3.98_ = 7.52, *P* = .002). At harvest (Day 35, after Rs1115 inoculation), RAL5 abundance in the consortium treatment was comparable to that in RAL5 single-strain treatment (*t*_2.48_ = −1.74, *P* = .199; Fig. 5C). In contrast, ACI4 showed lower mean densities under co-inoculation at all three sampling stages, but the between-treatment difference reached significance only at Day 7 (*t*_2.68_ = 3.45, *P* = .049). No significant differences were detected at Day 2 or harvest (*t*_2.39_ = 2.93, *P* = .081; *t*_2.01_ = 1.88, *P* = .200, respectively; Fig. 5D). Across all sampling points, although RAL5 maintained relatively higher population densities than ACI4 in line with the competitive asymmetry observed in vitro, ACI4 remained detectable throughout the greenhouse experiment, and the consortium ultimately yielded the strongest plant protection. Together, these results suggest that the emergent antagonism detected in vitro could translate into enhanced pathogen suppression also *in planta*, at least under the sterilized-soil conditions used here.

## Discussion

Interactions between bacterial strains can induce or suppress antagonistic activity against a target organism, resulting in inhibitory phenotypes that differ from those observed in monocultures [21, 22, 47]. In this study, RAL5 and ACI4 each showed little constitutive secreted inhibition of the phytopathogen *R. solanacearum* Rs1115, yet their interaction generated stronger pathogen suppression. The magnitude of pathogen suppression increased with the strength of reciprocal inhibition between the two resident strains. The interaction was asymmetric: RAL5 dominated the co-culture, whereas ACI4 declined and was strongly inhibited by the 48-h co-culture supernatant. Transcriptomic and metabolomic analyses further showed that the onset of pathogen suppression coincided with strongly asymmetric transcriptional responses in the dominant RAL5 and a profound chemical restructuring of the co-culture environment. These changes included the accumulation of several antimicrobial compounds, such as novobiocin- and tylosin B-like antibiotics and the benzenoid 4-aminophenol. In addition to confirming that these compounds inhibited Rs1115, we found that intracellular contents released upon ACI4 lysis when competing with RAL5 were also suppressive to the pathogen. The effects of the RAL5–ACI4 combination were not restricted to short-term in vitro assays but could be translated into improved biocontrol outcomes *in planta*. These findings highlight an additional layer of functional organization in microbial communities, where certain taxa contribute to invasion resistance not by directly inhibiting the pathogen, but by acting as ecological triggers that unlock otherwise dormant pathogen suppression within the plant rhizosphere microbiome [40, 48, 49].

Systematic co-culture screening identified only a limited subset of resident isolates capable of enhancing Rs1115 suppression when paired with RAL5 (6 of 51; Fig. 2A). Several of the enhanced hits belonged to the genus *Acinetobacter* (4 of 6), and similar enhancement was also observed when RAL5 was replaced by closely related non-pathogenic *Ralstonia* strains (Fig. S2). Although this pattern does not imply that all *Acinetobacter* isolates function as activating partners, it does suggest that interaction-mediated activation may be more common in certain taxonomic pairings than in others. This interpretation is consistent with previous high-throughput surveys showing that interaction-induced antimicrobial activity, although relatively rare (≈6%), is non-randomly distributed and often enriched within specific phylogenetic groups [50]. Studies in *Streptomyces* have shown that interaction-induced increases in inhibitory activity are more likely when partner strains are closely related, share secondary-metabolite BGCs, or have greater nutrient-use overlap [21, 51]. In our system, anthranilate depletion together with *antABC* induction in ACI4 points to a potential metabolic interaction between ACI4 and the *Ralstonia* background (Fig. 3D; Fig. S10), which may partly explain the observed effects between these taxonomic pairings. The anthranilate biosynthetic enzyme TrpEG is highly conserved (>85% identity) across the genus *Ralstonia*, and anthranilate is a well-characterized intraspecies signalling molecule that positively regulates virulence in the pathogen *R. solanacearum* [52, 53]. Although the RAL5–ACI4 pair suppressed Rs1115 in vitro and reduced bacterial wilt severity *in planta*, more research is needed to validate if anthranilate metabolism contributes to this observed pattern.

Untargeted metabolomics detected several candidate antimicrobial features underlying the emergent antagonism, including novobiocin- and tylosin B-like compounds and the benzenoid 4-aminophenol (Fig. 4C). Representative standards corresponding to novobiocin- and tylosin B-like annotations inhibited Rs1115, supporting their potential contribution to co-culture suppressiveness (Fig. 4D–F). However, these antibiotic-like features should be interpreted cautiously because complete KEGG-annotated biosynthetic pathways for these complex compounds were not detected in either the RAL5 or ACI4 genomes. Among the remaining candidate features, 4-aminophenol was identified with the highest confidence level and was strongly enriched during late stages of co-culturing (Fig. 4C). This low-molecular-weight redox-active compound is capable of undergoing spontaneous oxidation reactions and contributes to oxidative stress in biological systems [54]. No established BGC has yet been linked to 4-aminophenol production, and no dedicated bacterial enzymatic pathway has been reported. Its accumulation is therefore more consistent with a non-canonical aromatic derivative emerging from a stress-altered metabolic landscape than with a defined biosynthetic program. Co-culture was also accompanied by the accumulation of lysophospholipids, including lysophosphatidylcholines (LPCs; Fig. 4C). Such membrane-active lipids are known to increase membrane permeability and sensitize cells to chemical and oxidative stress, thereby potentially amplifying the impact of redox-active small molecules present in the extracellular milieu [55, 56]. RAL5 upregulated arylpolyene biosynthetic genes (Fig. S9), whose products protect membrane lipids from oxidative damage [57], suggesting an enhanced capacity for membrane protection in this chemically stressful environment. Consistent with this interpretation, RAL5 retained higher viability than ACI4 following direct exposure to 4-aminophenol (Fig. 4G). This difference in susceptibility may help explain the establishment of RAL5 dominance within the co-culture. However, targeted mutagenesis and complementation will be required in the future to determine which candidate genes and pathways are necessary for the emergent inhibitory phenotype, and to distinguish which cellular and molecular responses are involved in the production and release of inhibitory compounds. Collectively, these findings suggest that pathogen suppression reflects combined antimicrobial pressures generated during interspecific competition, including antibiotic-like activity, redox-active metabolites, and membrane-associated stress.

Compatibility among community members is widely regarded as a key design principle for synthetic microbial consortia, because strong reciprocal inhibition can drive competitive exclusion and thereby compromise consortium function [58, 59]. However, incompatibility or compatibility detected in vitro does not reliably predict *in planta* performance [60, 61]. In our system, intense competition between RAL5 and ACI4 caused a rapid population decline of ACI4 in homogeneous liquid co-cultures (Fig. 2C) and the activation of pathogen suppression depended on the strength of competition between RAL5 and ACI4 (Fig. 2D) and became weaker if the initial ACI4 frequency was low (Fig. S7). However, no ACI4 extinctions were observed in the rhizosphere, where the RAL5–ACI4 consortium consistently outperformed single-strain treatments by more strongly suppressing *R. solanacearum in planta* (Fig. 5A and B). One explanation for this is that spatial heterogeneity and micro-niche refuges likely buffered ACI4 against competitive exclusion [62], allowing ACI4 to persist and contribute to the suppressive effect of the consortium over a much longer time scale compared to in vitro assays (Fig. 5D). However, the greenhouse assays were conducted in sterilized soil, thereby minimizing competition and priority effects imposed by pre-established resident microbiota [63, 64]. In natural soils, indigenous communities may constrain inoculant establishment or alter the strength or sign of the RAL5–ACI4 interaction through higher-order effects [65, 66]. Future work should test whether the RAL5–ACI4 consortium inoculants can establish and persist in the presence of indigenous rhizobacteria, and whether the consortium retains its biocontrol efficacy under these natural conditions. Studies across different soils and microbial communities are also needed to determine how common the emergent antagonism is and whether effective partner strains depend on the composition of the background community.

From a broader perspective, this study illustrates how pathogen suppression can emerge as a by-product of pairing a non-pathogenic resident strain with another non-antagonistic partner strain that intensifies interspecific competition and may stimulate antimicrobial metabolite production. In our study, suppression arose through interaction-induced stress responses and combinatorial antimicrobial effects, including antibiotic-like metabolites, redox-active small molecules, and lysis-associated release of intracellular contents. This likely resulted in non-specific suppression targeting multiple cellular components, and may offer an evolutionarily robust barrier that limits pathogen resistance evolution compared to single-target antibiotic pressure [67]. These findings show that interaction-based screening can complement conventional isolate-centered biocontrol agent discovery by identifying suppressive functions that are not expressed in bacterial monocultures. Our study also highlights the need to investigate antimicrobial activity in taxa not traditionally regarded as antibiotic producers such as *Ralstonia* and *Acinetobacter*, where secondary metabolism or stress-associated defense responses might be activated only during interspecific competition. Although greenhouse assays confirmed the protective effect of the RAL5–ACI4 consortium *in planta*, our transcriptomic and metabolomic analyses were conducted in vitro and therefore did not resolve whether and how these interaction-induced responses occurred or changed during rhizosphere colonization. Microbial interactions can evolve over short timescales [68, 69], and heritable changes accumulating across successive generations could modify the RAL5–ACI4 interaction and its consequences for pathogen suppression. These limitations leave open whether the mechanisms identified in vitro also operate in the rhizosphere and whether interaction-dependent suppression persists beyond the duration of the greenhouse experiment. Harnessing microbial interactions to elicit biocontrol functions offers a complementary strategy for engineering disease-suppressive microbiomes, but its practical value will depend on whether the resulting suppression can be maintained under field conditions and over longer timescales.

## Supplementary Material

Supplementary_material_wrag229

## Data Availability

The whole-genome sequencing data for *Ralstonia pickettii* RAL5 and *A. oleivorans* ACI4, together with all RNA-seq data generated in this study, have been deposited in the NCBI Sequence Read Archive (SRA) under BioProject PRJNA1333583. RAL5 genome data are available under accession numbers SRR37675514 and SRR37675515; ACI4 genome data under SRR35576687 and SRR35576688; and RNA-seq data under SRR35576664–SRR35576681. Additional data and information supporting this study are provided in the supplementary information.
