# Supplementary material for: Competition between resident rhizosphere bacteria enhances phytopathogen suppression via emergent antagonism
Source: ISME J. 2026 Sep 4;20(1):wrag229. doi: 10.1093/ismejo/wrag229 (PMC13637822; doi:10.1093/ismejo/wrag229)
Supplement: Supplementary file 1 [file supplementary_material_wrag229.zip › Supplementary Information.pdf]

Supplementary Information for

Competition between resident rhizosphere bacteria enhances phytopathogen suppression via emergent antagonism

This file contains the following supplementary materials:

- Supplementary Methods
- Supplementary Table S2

  [Tables S1 and S3–S8 are provided in a separate Excel workbook]
- Supplementary Figures S1–S10
- Supplementary References

Supplementary Methods

Substitution assays with related non-pathogenic strains

To assess whether the partner-associated enhancement of pathogen suppression was specific to RAL5, four phylogenetically related non-pathogenic strains were used in place of RAL5 and co-cultured with one partner strain (*Acinetobacter oleivorans* ACI4), which was observed to induce improved pathogen suppression in a co-culture with RAL5. Co-cultures and corresponding monocultures were established under the same conditions used for the main RAL5-based co-culture screening, with a 1:1 inoculation ratio and a total initial inoculum of approximately 10^6 CFU mL^{-1}. After 48 h of incubation, cell-free supernatants were collected and tested against *R. solanacearum* Rs1115 using the same supernatant inhibition assay. Inhibition efficiency was calculated relative to the negative control, in which sterile 20% NB medium was used in place of bacterial supernatant. All assays were performed in three independent biological replicates.

Supernatant inhibition assays against Rs1115 and OP50

To determine the temporal emergence and frequency-dependence of inhibitory activity against *Ralstonia solanacearum* QL-Rs1115 (hereafter Rs1115), cell-free supernatants were collected from monocultures of RAL5 and ACI4 and from RAL5–ACI4 co-cultures. For temporal assays,

supernatants were collected at 12, 24, 36, 48, 60, and 72 h from 1:1 RAL5–ACI4 co-cultures and the corresponding monocultures. For frequency-dependence assays, RAL5–ACI4 co-cultures were established at initial RAL5:ACI4 ratios of 1:1, 1:10, 1:100, 10:1, and 100:1, with the total initial cell density maintained at approximately 10^6 CFU mL^{-1} across all treatments. Cell-free supernatants were collected after 48 h and tested against Rs1115 using the same inhibition assay as in the initial screen. Briefly, supernatants were added to 20% NB medium at 10% (v/v). Sterile 20% NB medium was used as the negative control, and Rs1115 growth was quantified by measuring OD_{600} after 24 h of incubation. Each treatment was performed in three independent biological replicates.

To assess whether inhibitory activity extended beyond Rs1115, *Escherichia coli* OP50 was used as an indicator strain. OP50 was cultured in LB at 37°C and prepared according to the main Methods. Cell-free supernatants collected at 48 h from monocultures of RAL5 and ACI4 and from their 1:1 co-culture were tested against OP50 using the same inhibition assay, except that OP50 cultures were incubated in 20% LB at 37°C. Each treatment was evaluated using 18 independent biological replicates.

Carbon utilization profiling and niche overlap analysis between RAL5, ACI4, and Rs1115

Carbon utilization assays were performed to characterize resource-use overlap between RAL5 and ACI4 and to compare both strains with the pathogen Rs1115. Each strain was inoculated individually into OS minimal medium [1] supplemented separately with 10 mM of each of 48 distinct carbon sources representative of tomato root exudates [2] (Table S5). Assays were performed in four independent biological replicates. After 48 h of incubation at 30°C, substrate utilization was scored as positive (1) when the mean net OD_{600} increase exceeded 0.05, and as negative (0) otherwise. Carbon utilization patterns were summarized using two indices: (i) niche breadth, defined as the total number of substrates utilized by a strain, and (ii) niche overlap, calculated as the proportion of shared substrates relative to the total number of substrates utilized by any strain.

Supernatant-mediated effects on RAL5 and ACI4

To assess the effects of RAL5–ACI4 co-culture supernatants on the two resident strains, cell-free supernatants were collected from 12 independent 48 h RAL5–ACI4 co-cultures and tested separately

against RAL5 and ACI4. For each assay, 20 µL of cell-free supernatant was mixed with 2 µL of recipient culture and 178 µL of fresh 20% NB medium in 96-well plates. Sterile 20% NB medium was used instead of supernatant as the negative control. Plates were incubated at 30°C with shaking at 170 rpm for 24 h. Growth inhibition was calculated as the percentage reduction in growth relative to the negative control.

To test whether either strain secreted compounds that inhibited the other resident strain, reciprocal supernatant assays were performed using monoculture supernatants. Cell-free supernatants were collected from four independent 48 h monocultures of each strain. RAL5 was exposed to ACI4-derived supernatants, and ACI4 was exposed to RAL5-derived supernatants using the same supernatant assay described above. Sterile 20% NB medium was used as the negative control. After 24 h of incubation at 30°C with shaking at 170 rpm, growth was quantified as OD600.

RNA extraction and transcriptomic library preparation

Total RNA was extracted from the frozen cell pellets using TRIzol reagent (Invitrogen, USA) according to the manufacturer's protocol. The extracted RNA was treated with DNase I (TaKaRa Bio, Japan) to remove genomic DNA, and RNA integrity was assessed using an Agilent 2100 Bioanalyzer. Ribosomal RNA (rRNA) was removed using the Ribo-Zero rRNA Removal Kit (Illumina, USA). Strand-specific paired-end libraries were prepared using the TruSeq RNA Sample Preparation Kit (Illumina, USA) prior to sequencing.

Genome sequencing, assembly, and annotation

To obtain high-quality and consistently annotated reference genomes for downstream transcriptomic and functional analyses, the genomes of *R. pickettii* RAL5 and *A. oleivorans* ACI4 were sequenced in this study. Genomic DNA of RAL5 and ACI4 was extracted from overnight cultures using the OMEGA Bacteria DNA Kit and quantified with a TBS-380 fluorometer (Turner BioSystems Inc., Sunnyvale, CA). High-quality DNA ($OD_{260/280}$=1.8–2.0, >6 µg) was used for library preparation. PacBio long-read sequencing on a Pacific Biosciences RS instrument and paired-end short-read sequencing on a NovaSeq 6000 System (Illumina) were performed by Shanghai Biozeron

Biotechnology Co., Ltd. Hybrid de novo assembly was performed using Unicycler v0.4.8 [3]. Gene prediction for both genomes was conducted using GeneMarkS v4.17 [4]. Predicted protein-coding sequences were functionally annotated against the NCBI non-redundant (NR) and Swiss-Prot databases using DIAMOND v0.9.22.123 [5]. KEGG orthologues were assigned using KofamScan v1.2.0 [6]. Secondary metabolite biosynthetic gene clusters were identified using antiSMASH v7.1.0 [7].

Untargeted LC–MS/MS metabolomic profiling

Cell-free supernatants collected from the RAL5 and ACI4 monocultures and co-cultures at 48 h were thawed on ice and vortexed briefly before extraction. For each sample, 50 μL of supernatant was mixed with 150 μL of extraction solvent (acetonitrile:methanol = 1:4, v/v) containing an internal standard. The mixtures were vortexed for 3 min and centrifuged at 12,000 rpm for 10 min at 4°C. An aliquot of 150 μL of the resulting supernatant was transferred to a clean tube, precipitated at −20°C for 30 min, and centrifuged again at 12,000 rpm for 3 min to remove residual particulates. A final volume of 120 μL of clarified extract was used for LC–MS analysis.

Untargeted LC–MS/MS acquisition was conducted on a Waters ACQUITY UPLC system equipped with an HSS T3 C18 column (1.8 μm, 2.1 mm × 100 mm) maintained at 40°C. The mobile phases consisted of water with 0.1% formic acid (A) and acetonitrile with 0.1% formic acid (B) delivered at a flow rate of 0.40 mL min^{-1}. Chromatographic separation followed a linear gradient from 5% to 90% B over 11 min, held for 1 min, returned to 5% B within 0.1 min, and re-equilibrated for 1.9 min. Samples were analysed in both positive and negative electrospray ionization modes.

Raw LC–MS files were converted to mzML format using ProteoWizard and processed with XCMS for feature detection, retention-time alignment, and grouping. Signal drift was corrected using a support vector regression (SVR) algorithm, and features detected in fewer than 50% of samples within any group were removed. Metabolite annotation was conducted using the annotation workflow provided by Wuhan Metware Biotechnology Co., Ltd. Annotated features were classified into four confidence levels. Level 1 metabolites were identified by matching retention times and MS/MS spectra to an in-house library of authentic standards. Level 2 annotations were assigned based on spectral

matching with integrated public databases. Level 3 annotations were derived from AI-assisted prediction or MetDNA analysis. Level 4 denoted unknown features lacking valid annotations which were excluded from further analysis.

Agar spot assay for novobiocin susceptibility

To compare the susceptibility of RAL5, ACI4, and Rs1115 across a novobiocin concentration gradient, agar spot assays were performed on NA plates. Bacterial lawns were prepared by spreading 200 μL of each strain (OD_{600} = 0.50) onto the agar surface. Novobiocin solutions at 50, 25, 10, and 5 mg/mL were prepared in sterile water, and 5 μL of each solution was spotted onto the plates. After incubation at 30°C for 48 h, inhibition zones were recorded. The assay was performed with four biological replicates per treatment.

Rhizosphere sampling, DNA extraction, and strain-specific primer design

For rhizosphere sampling, loosely adhering soil was removed by gentle shaking, and tightly adhering rhizosphere soil was collected, immediately frozen in liquid nitrogen, and stored at −80°C. DNA was extracted from 0.5 g of soil using the NucleoSpin Soil Kit (MACHEREY-NAGEL, Germany) according to the manufacturer's instructions.

Strain-specific primers for RAL5 and ACI4 were designed to target unique single-copy marker genes. The proteomes of RAL5, ACI4, and Rs1115 were clustered into orthogroups using OrthoFinder v2.5.5 [8]. Target-specific single-copy genes absent from the Rs1115 genome were selected as candidate markers. Primer pairs were designed using Primer Premier v6.0 (Premier Biosoft International, USA) and validated for specificity using genomic DNA from non-target strains and soil background controls (Table S2).

Quantitative PCR standard curves and amplification parameters

Standard curves were generated using linearized recombinant pUC19 plasmids carrying the corresponding target gene fragments. Plasmids were purified and serially diluted over six orders of magnitude. Copy numbers were calculated from DNA concentration and fragment size. qPCR was

performed on a QuantStudio 3 Real-Time PCR System (Applied Biosystems, Thermo Fisher Scientific, USA) using PowerUp SYBR Green Master Mix. Each 20 μL reaction contained 10 μL of 2× Master Mix, 2 μL of soil DNA template, and 0.4 μL of each primer (10 μM). The qPCR program included an initial denaturation step at 95°C for 30 s, followed by 40 cycles of 95°C for 5 s and 60°C for 34 s. Melt curve analysis was conducted with the following steps: 95°C for 15 s, 60°C for 1 min, and 95°C for 15 s to confirm amplification specificity. All reactions were performed in technical triplicate. Bacterial abundances were calculated as gene copy numbers per gram of soil based on the corresponding standard curves.

Supplementary Table

Table S2. Strain-specific qPCR primers used in this study.

Bacterial strain	Primer type	Target gene	Primer sequence (5′–3′)	Origin
R. pickettii RAL5	Forward	*amiE*	CGCCGCCTGGAACAACATCTA	This study
	Reverse		TCAGTGCCGAGTTGCTGGAAG	This study
A. oleivorans ACI4	Forward	*vgrG*	GCAGGCGGCTCTCAAGTCAAA	This study
	Reverse		TGGCAACGCAGGAACATTAGCA	This study
R. solanacearum Rs1115	Forward	*fliC*	GAACGCCAACGGTGCGAACT	[9]
	Reverse		GGCGGCCTTCAGGGAGGTC	

Supplementary Figures

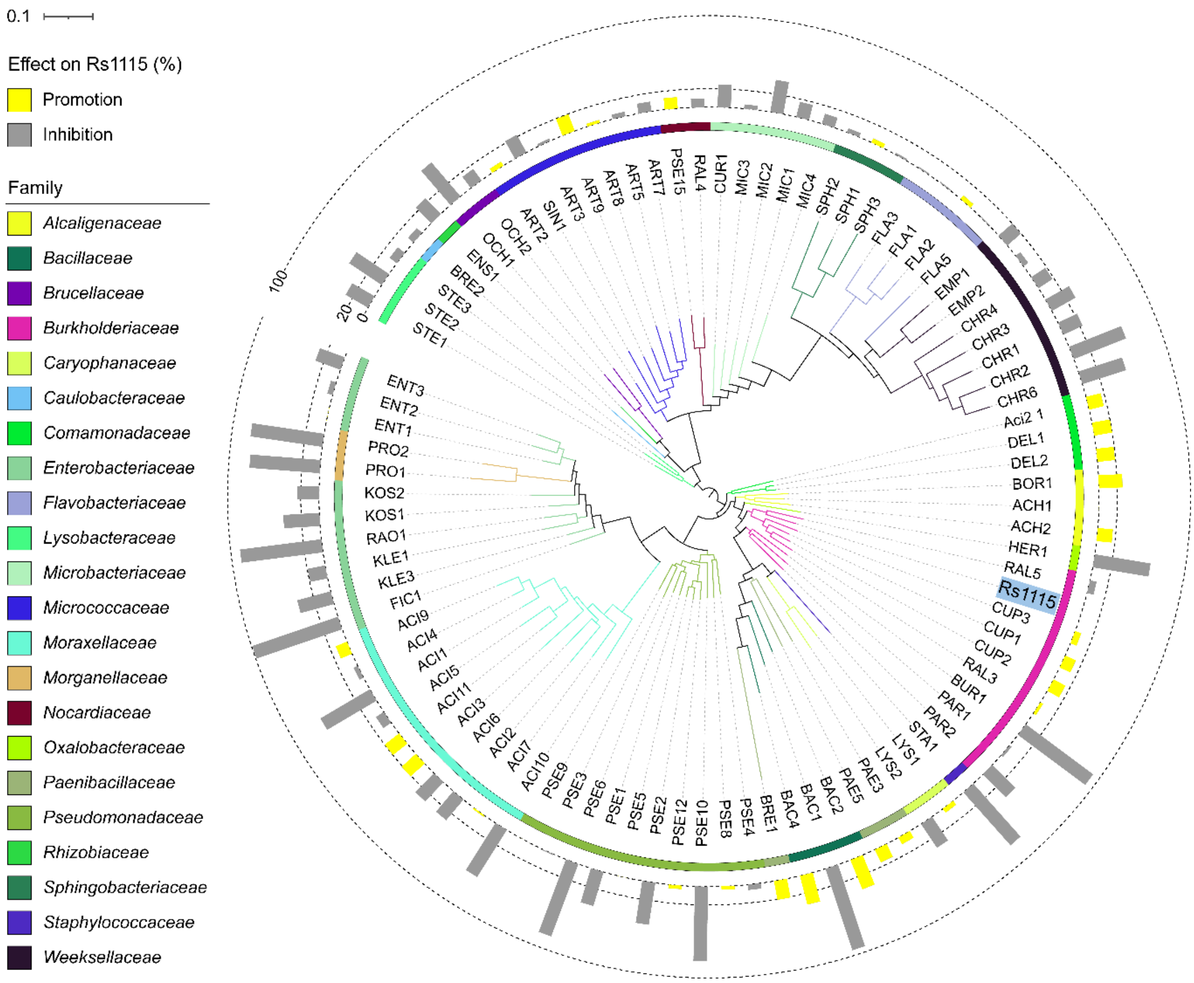

Figure S1. Whole-genome phylogenomic tree and supernatant effects of bacterial isolates on *Ralstonia solanacearum* Rs1115. The circular tree provides the genome-based phylogenomic context of *R. pickettii* RAL5, *R. solanacearum* Rs1115, and 89 resident tomato rhizosphere isolates representing 22 families and 37 genera (Table S1). The scale bar represents a Mash distance of 0.1. The outermost bar track shows the effects of cell-free supernatants from individual strains on Rs1115 growth. Supernatants were collected from 48 h monocultures and grey bars indicate inhibition, whereas yellow bars indicate the promotion of Rs1115 growth. Effects within ±20% were classified as weak effects.

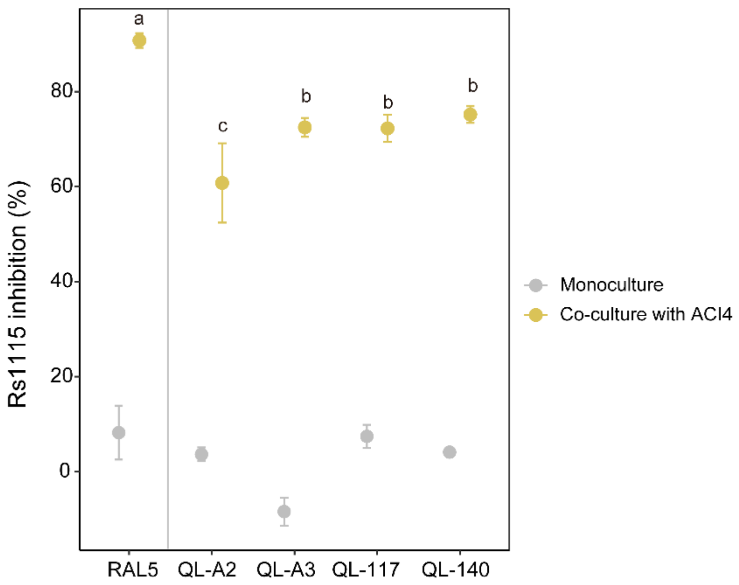

Figure S2. Inhibitory effects of supernatants produced by individual non-pathogenic *Ralstonia*-related isolates or by their co-cultures with ACI4 against *R. solanacearum* Rs1115. Grey dots represent inhibition by supernatants derived from monocultures of the individual strain, whereas yellow dots represent inhibition by supernatants derived from the corresponding co-cultures with ACI4. Error bars represent the mean ± SD. Differences in pathogen inhibition among co-culture treatments were analysed using one-way ANOVA, followed by Tukey's HSD test. Different letters indicate statistically significant differences ($P < 0.05$).

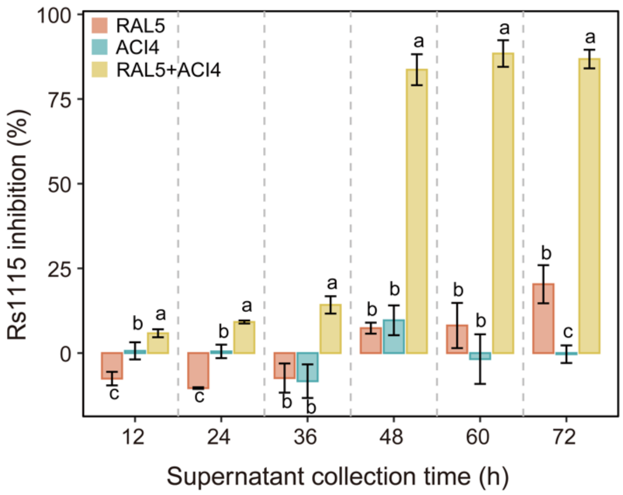

Figure S3. Pathogen inhibition by cell-free supernatants collected from different treatments over time (12–72 h). Inhibitory effects of supernatants derived from RAL5, ACI4, and the RAL5–ACI4 co-culture were measured at six time points. At each time point, different letters indicate statistically significant differences among treatments based on one-way ANOVA followed by Tukey's HSD test ($P < 0.05$). Error bars represent the mean ± SD of all replicates.

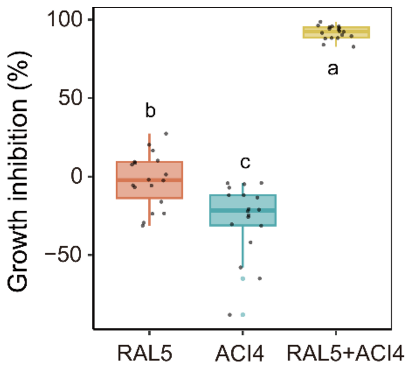

Figure S4. Inhibition of *E. coli* OP50 by cell-free supernatants collected from different treatments at 48 h. Each dot represents an individual measurement. Different letters indicate statistically significant differences among treatments based on the Kruskal–Wallis test followed by Dunn's test ($P < 0.05$).

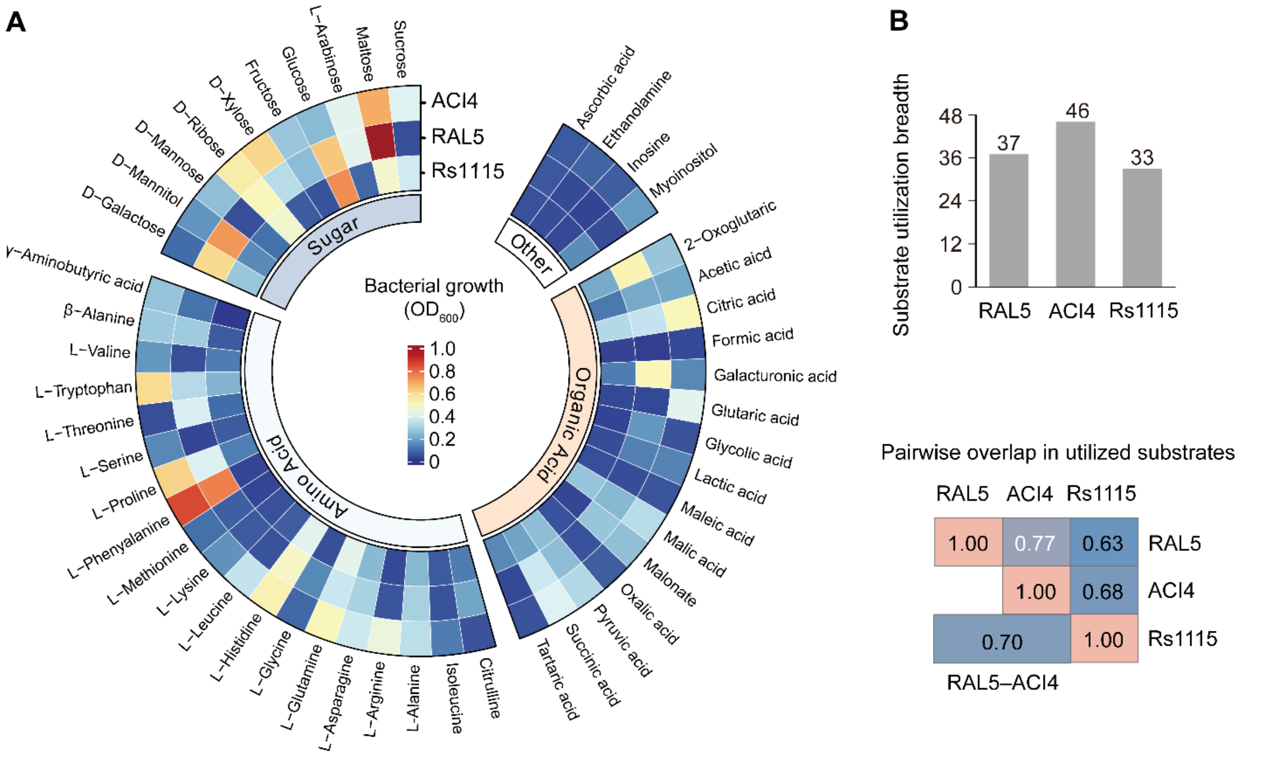

Figure S5. Carbon utilization patterns of RAL5, ACI4, and Rs1115. (A) Carbon utilization profiles of RAL5, ACI4 and Rs1115 across 48 representative carbon substrates. The circular heatmap shows growth on individual substrates, grouped into sugars, amino acids, organic acids, and other compounds. **(B)** Carbon niche breadth and pairwise niche overlap among the three strains. The bar plot shows the number of utilized substrates for each strain, and the matrix shows pairwise overlap in substrate utilization, with higher values indicating greater potential for resource competition.

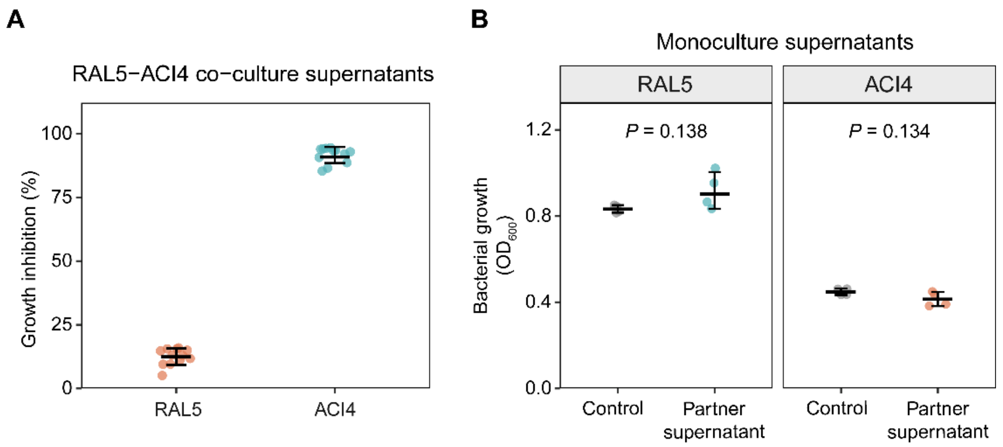

Figure S6. Supernatant-mediated effects between RAL5 and ACI4. (A) Effects of RAL5–ACI4 co-culture supernatants on RAL5 and ACI4. Inhibition was calculated relative to the negative control. **(B)** Reciprocal monoculture supernatant effects on the growth of ACI4 and RAL5. Growth was measured as OD_{600} after 24 h with or without exposure to partner-derived cell-free supernatant. In panel **(B)**, *P* values were calculated using Welch's t-test. In both panels, each point represents one biological replicate. Black horizontal bars indicate the mean, and error bars represent ± SD of biological replicates.

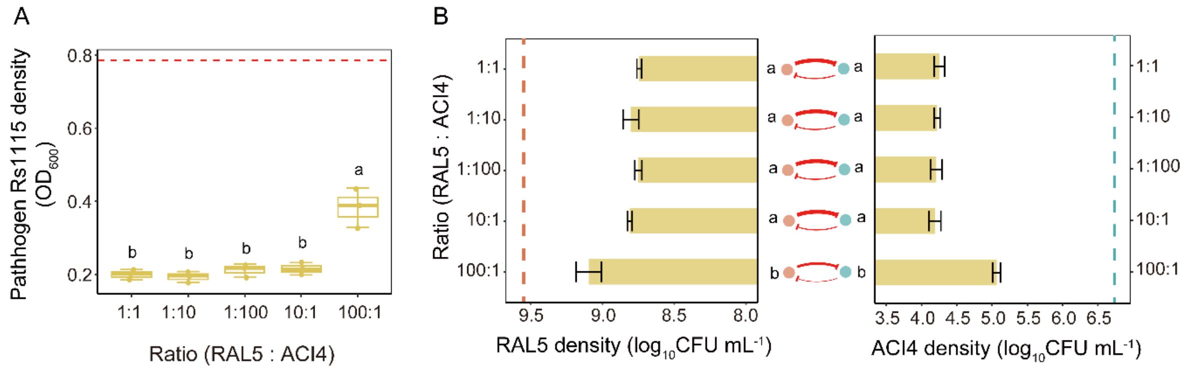

Figure S7. Frequency-dependent effects of the RAL5–ACI4 co-culture on pathogen inhibition and competitive interactions. (A) Effect of supernatants collected from RAL5–ACI4 co-cultures established at five different initial inoculation ratios on the growth of *R. solanacearum* Rs1115. The red dashed line indicates the pathogen-only control. **(B)** Population dynamics and competitive interactions between RAL5 and ACI4. Cell densities of RAL5 and ACI4 in monoculture (orange and teal dashed lines, respectively) and in co-culture at 48 h are shown across the five initial inoculation

ratios. The central interaction diagram illustrates the relative intensity of competition between the two strains. In both panels, different letters indicate statistical significance at $P < 0.05$ (one-way ANOVA followed by Tukey's HSD test). Error bars represent the mean ± SD.

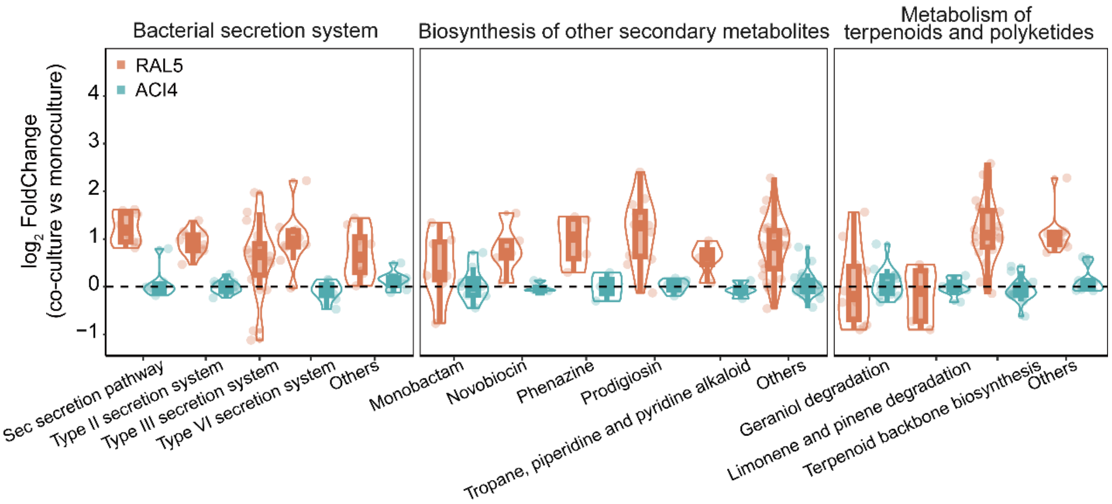

Figure S8. Transcriptional changes in secretion and secondary metabolism pathways. Differential gene expression (log_2FoldChange) in RAL5 and ACI4 under co-culture relative to their respective monocultures. Each dot represents an individual gene annotated to the corresponding KEGG pathways. Pathways containing three or fewer genes were aggregated into the "Others" category.

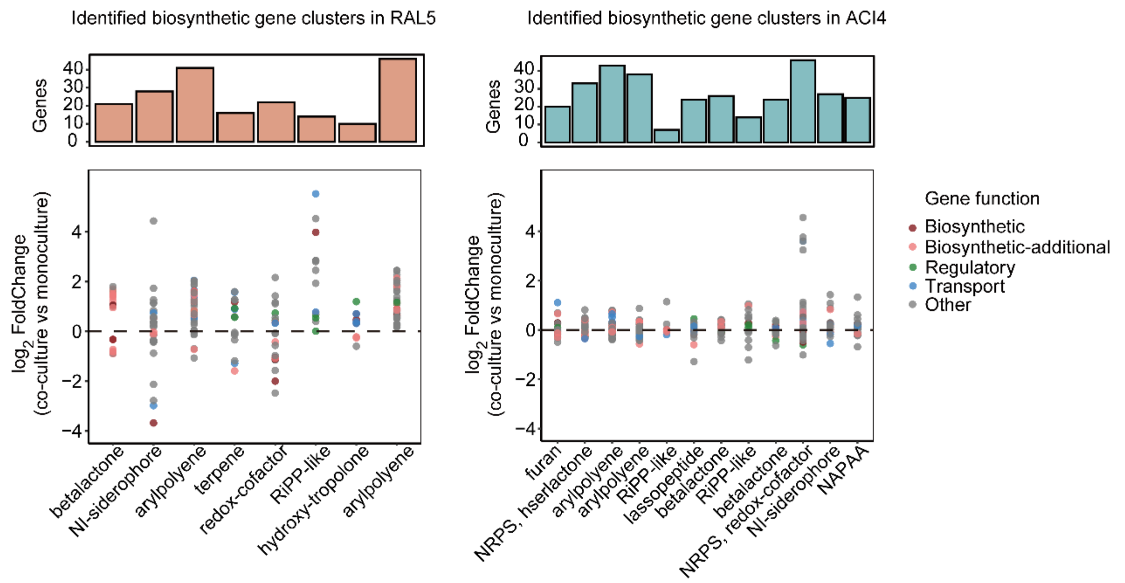

Figure S9. Expression changes of genes within biosynthetic gene clusters predicted by antiSMASH in RAL5 and ACI4 under monoculture and co-culture conditions. The upper bar chart displays the total gene count in each cluster. Each point represents an individual gene within these clusters, coloured according to its predicted functional annotation.

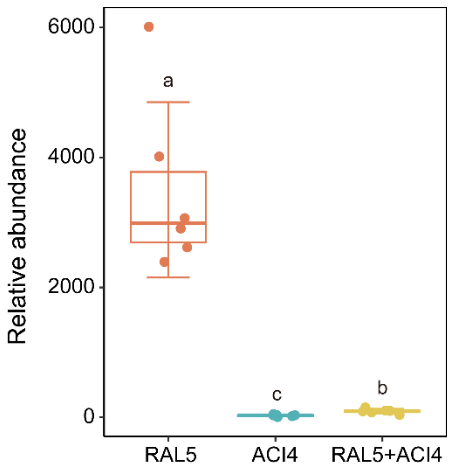

Figure S10. Relative abundance of anthranilic acid across different treatments. Anthranilic acid accumulation was highest in the RAL5 monocultures, whereas its abundance in the ACI4 monocultures and the RAL5–ACI4 co-cultures was close to zero. Individual points represent six biological replicates. Different letters indicate statistically significant differences among treatments (Kruskal–Wallis test followed by Dunn's test, $P < 0.05$).

Supplementary References

1. Schnider-Keel U, Seematter A, Maurhofer M *et al.* Autoinduction of 2,4-diacetylphloroglucinol biosynthesis in the biocontrol agent *Pseudomonas fluorescens* CHA0 and repression by the bacterial metabolites salicylate and pyoluteorin. *J Bacteriol* 2000;**182**:1215–25. https://doi.org/10.1128/JB.182.5.1215-1225.2000.

2. Wei Z, Yang T, Friman V-P *et al.* Trophic network architecture of root-associated bacterial communities determines pathogen invasion and plant health. *Nat Commun* 2015;**6**:8413. https://doi.org/10.1038/ncomms9413.

3. Wick RR, Judd LM, Gorrie CL *et al.* Unicycler: resolving bacterial genome assemblies from short and long sequencing reads. *PLoS Comput Biol* 2017;**13**:e1005595. https://doi.org/10.1371/journal.pcbi.1005595.

4. Besemer J, Lomsadze A, Borodovsky M. GeneMarkS: a self-training method for prediction of gene starts in microbial genomes. Implications for finding sequence motifs in regulatory regions. *Nucleic Acids Res* 2001;**29**:2607–18. https://doi.org/10.1093/nar/29.12.2607.

5. Buchfink B, Xie C, Huson DH. Fast and sensitive protein alignment using DIAMOND. *Nat Methods* 2015;**12**:59–60. https://doi.org/10.1038/nmeth.3176.

6. Aramaki T, Blanc-Mathieu R, Endo H *et al.* KofamKOALA: KEGG Ortholog assignment based on profile HMM and adaptive score threshold. *Bioinformatics* 2020;**36**:2251–2. https://doi.org/10.1093/bioinformatics/btz859.

7. Blin K, Shaw S, Augustijn HE *et al.* antiSMASH 7.0: new and improved predictions for detection, regulation, chemical structures and visualisation. *Nucleic Acids Res* 2023;**51**:W46–50. https://doi.org/10.1093/nar/gkad344.

8. Emms DM, Kelly S. OrthoFinder: phylogenetic orthology inference for comparative genomics. *Genome Biol* 2019;**20**:238. https://doi.org/10.1186/s13059-019-1832-y.

9. Schönfeld J, Heuer H, van Elsas JD *et al.* Specific and sensitive detection of *Ralstonia solanacearum* in soil on the basis of PCR amplification of *fliC* fragments. *Appl Environ Microbiol* 2003;**69**:7248–56. https://doi.org/10.1128/AEM.69.12.7248-7256.2003.
